# Molecular Diode‐Based Covalent Organic Frameworks: Imine Orientation‐Driven Acid‐Base Switching Photocatalytic H_2_ Production

**DOI:** 10.1002/advs.202522414

**Published:** 2026-01-05

**Authors:** Tianyi Liu, Yunjie Lang, Ning Sun, Xu Fang, Jinhua Rao, Yuchun Xu, Zhen Li, Weiqiao Deng

**Affiliations:** ^1^ Institute of Frontier Chemistry School of Chemistry and Chemical Engineering Shandong University Qingdao Shandong China; ^2^ School of Integrated Circuits Ludong University Yantai Shandong China

**Keywords:** covalent organic frameworks, isomeric structures, molecular diodes, photocatalytic hydrogen production, pH‐responsive rectification

## Abstract

Donor–acceptor type covalent organic frameworks (D–A COFs) have emerged as a promising class of photocatalytic materials due to their highly porous structures and excellent photo charge separation. However, the role of linkage between donor and acceptor in regulating charge transport and reaction selectivity remains not fully elucidated. Inspired by molecular diodes with similar D–A structure and specific rectification character, we designed and synthesized two molecular diode‐based COF isomers (PyAm‐PhAl‐COF and PyAl‐PhAm‐COF), and systematically investigated their switchable electron transfer and photocatalytic hydrogen evolution. These isomers exhibit pronounced pH‐responsive current rectification during photo‐induced electron transfer, with the behavior directly driven by imine bond orientation. Specifically, PyAm‐PhAl‐COF facilitates efficient electron transfer under alkaline conditions, achieving a hydrogen evolution rate 172 times higher than that under acidic conditions. In contrast, PyAl‐PhAm‐COF displays an opposite trend, with a 25‐fold activity enhancement under acidic vs. alkaline environments. This “acid‐base switching effect” originates from protonation/deprotonation‐induced reversal of the imine bond dipole: the dipole change dynamically regulates the intramolecular electron transport pathway, thereby governing the selective oxidation of different electron donors. These findings not only deepen the understanding of structure‐performance relationships in D–A COFs but also provide a new design strategy for developing adaptive smart photocatalytic systems.

## Graphical abstract

Molecular diode‐based covalent organic frameworks (COFs) enable pH‐responsive current rectification, dynamically reversing electron flow to toggle photocatalytic hydrogen evolution activity between acidic and alkaline environments.

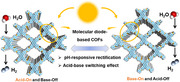

## 1 Introduction

Covalent organic frameworks (COFs) have emerged as promising photocatalyst materials, garnering significant attention over the past decade [1]. Their excellent porosity and large specific surface area provide abundant space for surface‐catalyzed reactions [2, 3, 4, 5, 6]. Furthermore, their structural designability and atomic‐level controllability offer exceptional potential for tuning critical factors in photocatalysis, including light absorption, energy band structure, exciton dissociation, charge transfer, and reactive site construction [7, 8, 9, 10]. To date, thousands of novel COFs have been developed for photocatalytic hydrogen evolution research, with several demonstrating remarkably high H_2_ evolution rates [11, 12, 13, 14, 15, 16, 17, 18].

The construction of donor‐acceptor (D–A) COFs is a commonly used strategy for developing efficient COF photocatalysts [19, 20]. The potential difference between the donor and acceptor serves as the driving force for the effective transfer and separation of photogenerated charge carriers [21, 22, 23, 24]. At the same time, since the donor and acceptor act as two ligands that form the extended structure of the COF, it is necessary to establish an effective connection between them to ensure pathways for electron transfer [25]. Therefore, the linkers between the donor and acceptor play an important role in the transfer of photogenerated charge carriers [26]. The orientation of the polar imine bond has emerged as a critical design parameter beyond the linkage itself, fundamentally governing charge dynamics. Thomas and co‐workers introduced the critical design principle of linkage orientation isomerism, showing the consistent superiority of the DCNA structures over the DNCA structures for hydrogen evolution [27]. This understanding was quantitatively refined by Zhao and co‐workers, who uncovered a fundamental trade‐off between exciton binding energy and charge transfer efficiency, modulated by the C═N bond polarity, with an optimum at intermediate polarity [28]. Most recently, Li and co‐workers showed an approach, achieving unprecedented performance by elucidating and harnessing the synergy between linker length, imine orientation, and protonation. However, most studies on isomeric COFs have been conducted under identical reaction conditions, while investigations into how charge separation, transport, and catalytic performance vary across multiple conditions remain scarce yet critically important [29, 30].

Similar to the D–A strategy in COFs, molecular diodes also utilize analogous D–A frameworks [31, 32]. This paradigm of intrinsic molecular asymmetry for rectification originated with the pioneering Aviram–Ratner model in 1974, which conceptualizes a structure centered on an electron donor and acceptor linked by a saturated σ‐bridge. This built‐in structural asymmetry enables the molecule to exhibit an externally tunable rectifying effect, namely, highly efficient directional electron transfer under certain forward conditions [33, 34, 35, 36, 37]. Thus, incorporating molecular diodes into the extended structure of COFs to enhance the transport of charge carriers under photoexcitation during photocatalysis, via the rectification effect of molecular diodes, represents a potential strategy for developing high‐performance COF‐based photocatalysts [38].

Here, we constructed two types of molecular diode‐based COF structures as photocatalysts, using imine bonds as the linking units to connect the donor and acceptor building blocks. We found that these two molecular diode‐based COF structures exhibit pronounced pH‐responsive rectification characteristics in photo‐induced electron transfer. This is primarily due to the influence of protonation and deprotonation of the imine bonds on the direction of electron transfer. For PyAl‐PhAm‐COF, electron transfer from donor to acceptor is blocked under neutral and alkaline conditions but is enabled under acidic conditions. In contrast, for PyAm‐PhAl‐COF, electron transfer from donor to acceptor is enabled under neutral and alkaline conditions but blocked under acidic conditions. This results in opposite photocatalytic hydrogen evolution performances for the two COFs under acidic and alkaline conditions in the presence of hole sacrificial agents. For PyAm‐PhAl‐COF, the hydrogen evolution rate under alkaline conditions is 172 times that under acidic conditions. For PyAl‐PhAm‐COF, the hydrogen evolution rate under acidic conditions is 25 times that under alkaline conditions. Fundamental characterization revealed that pristine PyAm‐PhAl‐COF possesses superior charge separation and transfer capabilities compared to PyAl‐PhAm‐COF. Interaction with acidic or alkaline sacrificial agents induced distinct alterations in charge carrier behavior within the COFs, leading to selective oxidation of the respective donors. Interactions with acidic or alkaline sacrificial agents lead to significant alterations in charge carrier behavior within the COF, resulting in pH‐responsive rectification characteristics. Consequently, the two COFs exhibit diametrically opposed hydrogen evolution activities under acidic and alkaline conditions. This work provides a new material platform and design strategy for developing tunable and pH‐responsive smart photocatalytic systems.

## 2 Results and Discussion

**FIGURE 1:**
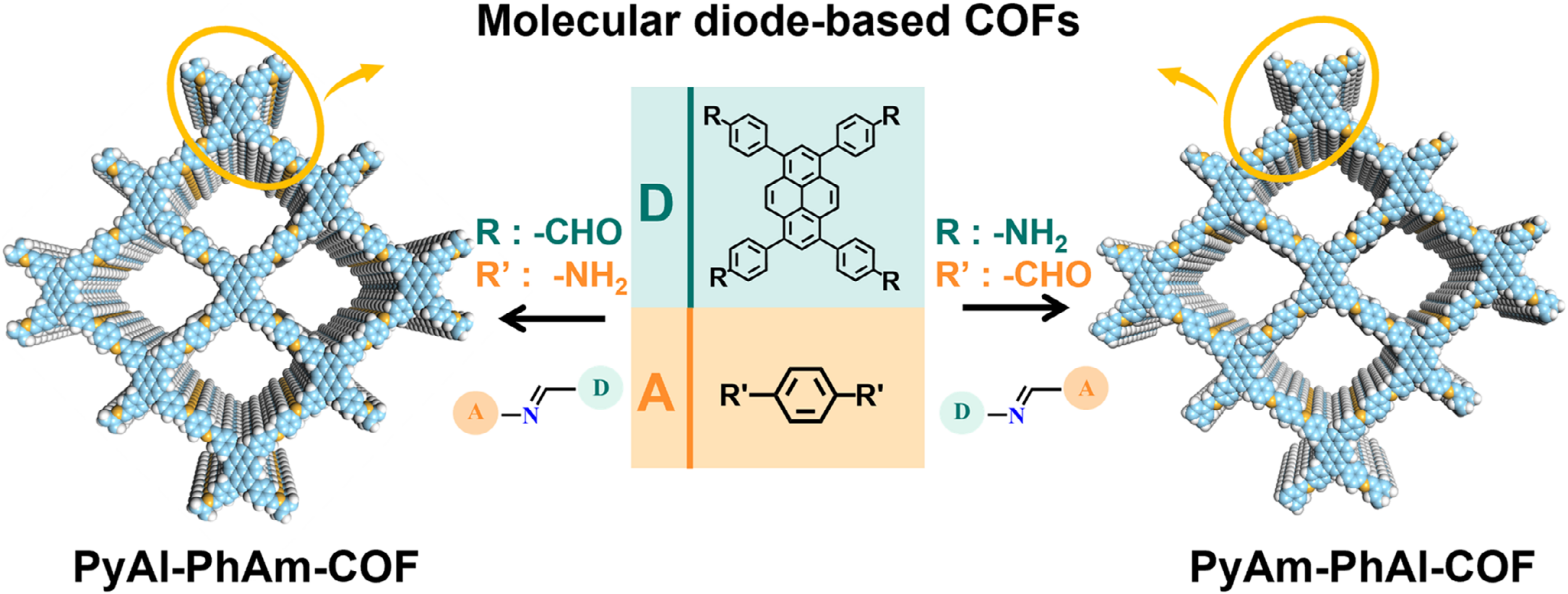
Synthesis processes and structures of PyAm‐PhAl‐COF and PyAl‐PhAm‐COF.

Two donor–acceptor (D–A) COF isomers, denoted as PyAm‐PhAl‐COF and PyAl‐PhAm‐COF, were synthesized through a Schiff base condensation reaction. The synthesis employed 1,3,6,8‐tetra(4′‐aldehydephenyl)pyrene (PyAl) with p‐phenylenediamine (PhAm), and 1,3,6,8‐tetrakis(4‐aminophenyl)pyrene (PyAm) with terephthalaldehyde (PhAl), respectively. The reaction was conducted in a mixed solvent system of n‐butanol (n‐BuOH) and o‐dichlorobenzene (o‐DCB) under acetic acid catalysis (Figures S1 and S2) [39, 40]. The key structural difference between the two COFs lies in the orientation of the imine bond: PyAm‐PhAl‐COF adopts a D─N═C─A configuration, whereas PyAl‐PhAm‐COF features a D─C═N─A arrangement (Figure 1).

**FIGURE 2:**
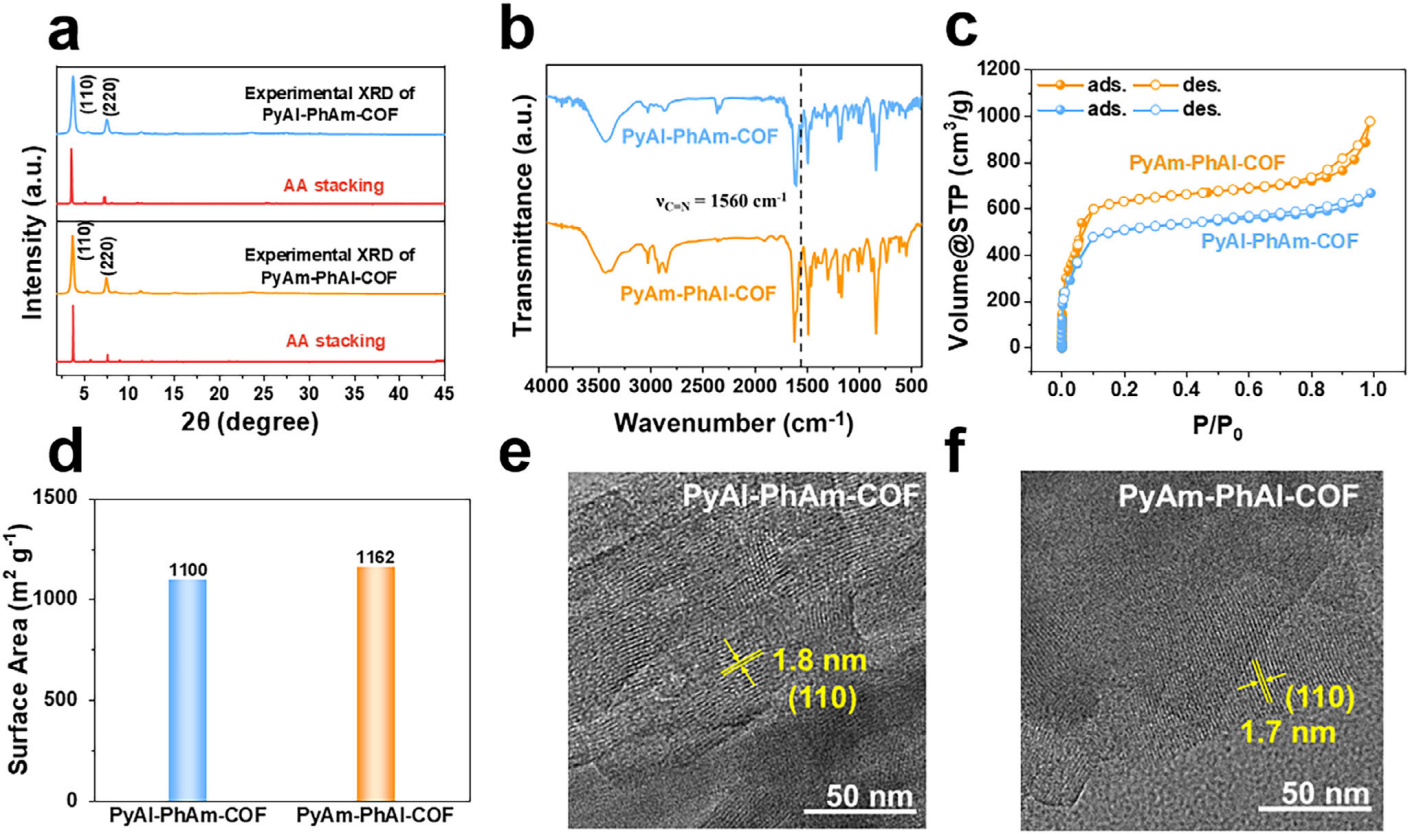
(a) Comparison of the experimental XRD patterns of the COFs and the corresponding simulated XRD of AA stacking structure. (b) FT‐IR spectra of PyAl‐PhAm‐COF (blue) and PyAm‐PhAl‐COF (orange). (c) N_2_ adsorption and desorption isotherms of COFs. (d) Specific surface area of the COFs. (e) HR‐TEM image for PyAl‐PhAm‐COF. (f) HR‐TEM image for PyAm‐PhAl‐COF. (Scale bar, 50 nm).

The crystalline structures of these COFs were determined by powder X‐ray diffraction (PXRD). Significant diffraction peaks corresponding to the (100) and (200) planes were observed for both PyAm‐PhAl‐COF and PyAl‐PhAm‐COF (Figure 2a). The experimental PXRD patterns closely matched the simulated PXRD patterns of the AA stacking model. The presence of imine linkages within the COFs was confirmed using Fourier‐transform infrared spectroscopy (FT‐IR) and ^13^C cross‐polarization magic angle spinning nuclear magnetic resonance spectroscopy (^13^C CP‐MAS NMR). The FT‐IR spectra of COFs showed the disappearance of the characteristic stretching signals for aldehyde (ν_C═O_ = 1692 cm^−1^) and amine (ν_N–H_ = 3352 cm^−1^) groups in the building blocks, along with the emergence of imine stretching signals (ν_C─N_ = 1560 cm^−1^) (Figure 2b; Figure S3), indicating successful condensation between aldehyde and amine groups to form imine linkages. The successful formation of the imine‐linked framework was further confirmed by solid‐state ^13^C NMR spectroscopy (Figure S4). The spectrum exhibited a distinct signal at 158 ppm, which corresponded to the characteristic fingerprint of the imine carbon (C═N). Furthermore, the signals observed in the range of 120–160 ppm were attributed to the aromatic carbons of the pyrene and phenyl building blocks, thus corroborating that these structural units remained intact within the polymerized architecture.

Thermogravimetric analysis (TGA) was performed to assess the thermal stability of the COF samples. The results indicated that PyAl‐PhAm‐COF remained stable below 300°C, while PyAm‐PhAl‐COF was stable up to 500°C (Figure S5). This might be attributed to the influence on interlayer interactions of 2D COFs by inverse imine bond orientations. For PyAm‐PhAl‐COF with D─N═C─A configuration, the direct connection of electron‐rich N^δ−^ to the donor pyrene enhances the electron density on the pyrene units, facilitating orbital overlap during interlayer stacking, thereby generating stronger interlayer π‐π interactions [41, 42, 43]. The COFs maintained high crystallinity even under exposure to different solvents such as THF, acetonitrile, 1 m NaOH, methanol, DMF, and water, underscoring their exceptional structural robustness (Figure S6). The porous nature of the COFs was investigated by nitrogen adsorption isotherms at 77 K. The adsorption curves of all COF materials confirmed their permanent porosity and exhibited typical type I behavior (Figure 2c). The Brunauer‐Emmett‐Teller (BET) specific surface areas of the samples were measured to be 1100 m^2^ g^−1^ for PyAl‐PhAm‐COF and 1162 m^2^ g^−1^ for PyAm‐PhAl‐COF, respectively (Figure 2d; Table S1). Non‐local density functional theory (NLDFT) analysis revealed nearly identical average pore sizes of 1.7 nm for both materials (Figure S7 and Table S1) [44]. The morphology of the COFs was examined using scanning electron microscopy (SEM) and high‐resolution transmission electron microscopy (HR‐TEM). The SEM images showed that the 2D COFs were aggregated in the form of particles and rods (Figure S8). The HR‐TEM images showed periodic backbones and lattice fringes, confirming the high crystallinity of the COFs (Figure 2e,f). In addition, the water adsorption and desorption curves verified that the two COFs had similar water adsorption capacity and wettability (Figure S9) [45].

**FIGURE 3:**
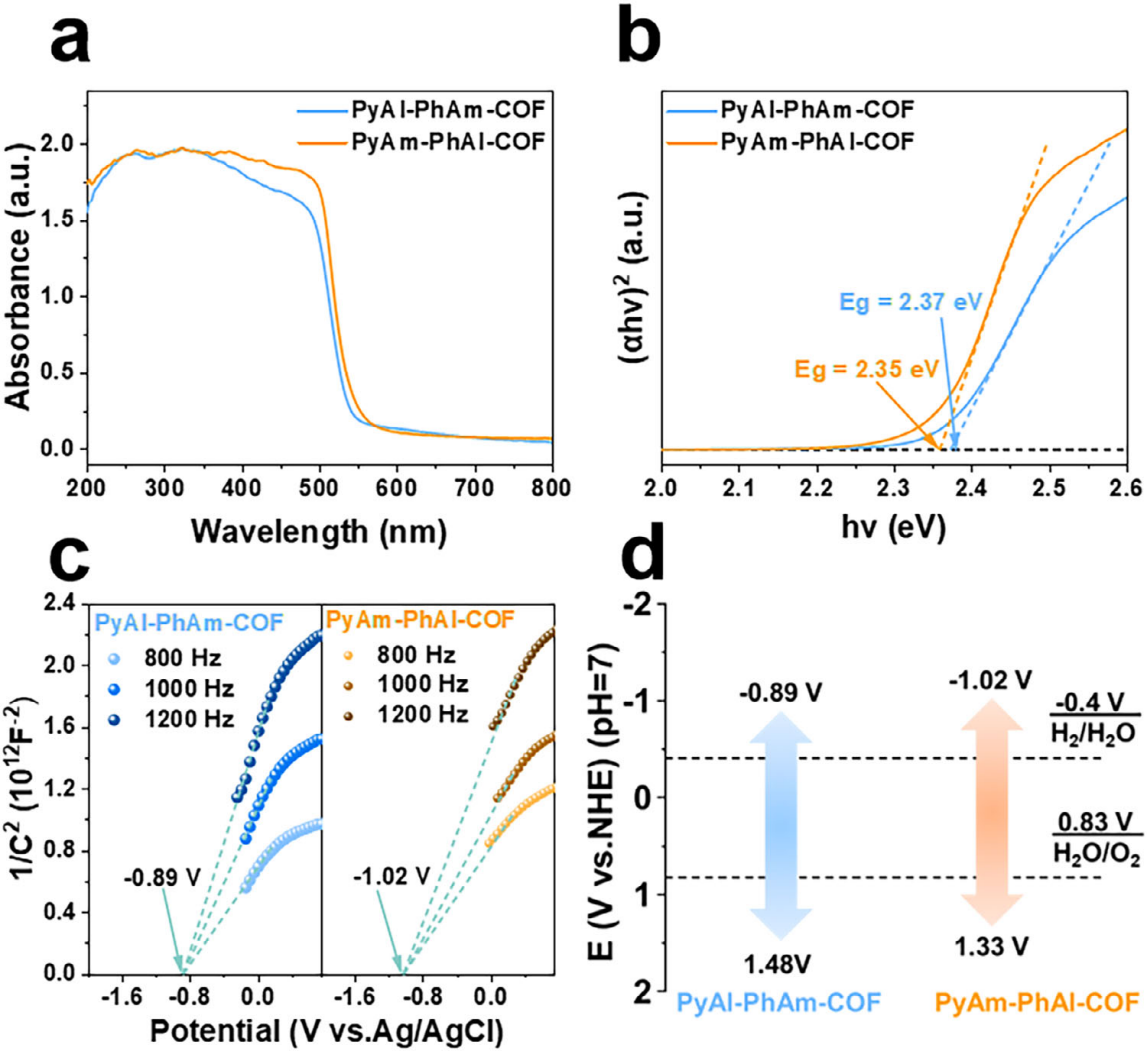
(a) UV–vis spectra of PyAl‐PhAm‐COF and PyAm‐PhAl‐COF. (b) Tauc plots of PyAl‐PhAm‐COF and PyAm‐PhAl‐COF. (c) Mott–Schottky plots of PyAl‐PhAm‐COF and PyAm‐PhAl‐COF. (d) Band structure of PyAl‐PhAm‐COF and PyAm‐PhAl‐COF.

To evaluate the potential of these COFs for photocatalysis, their light absorption properties and energy band structures were investigated by optical and electrochemical techniques. Ultraviolet‐visible (UV–vis) diffuse reflectance spectra showed that the COFs had similar light absorption profiles, absorbing light from the UV region to the visible region with an absorption edge of approximately 510 nm (Figure 3a). Correspondingly, the optical band gaps of the two COFs were calculated based on the Tauc curves to be 2.37 eV for PyAl‐PhAm‐COF and 2.35 eV for PyAm‐PhAl‐COF (Figure 3b). The Mott‐Schottky curves of both COFs showed positive slopes, indicating that they exhibited typical characteristics of n‐type semiconductors. The conduction band (CB) potentials were determined to be −0.89 and −1.02 V (vs. NHE) for PyAl‐PhAm‐COF and PyAm‐PhAl‐COF, respectively (Figure 3c). Accordingly, the valence band (VB) potentials were calculated to be 1.48 and 1.33 V (vs. NHE) for PyAl‐PhAm‐COF and PyAm‐PhAl‐COF, respectively. The corresponding energy band diagrams are shown in Figure 3d. In addition, the VB potentials were also measured by ultraviolet photoelectron spectroscopy (UPS), giving values of 1.51 and 1.36 eV (vs. NHE) for PyAl‐PhAm‐COF and PyAm‐PhAl‐COF, respectively. Based on these UPS results, the CB of PyAl‐PhAm‐COF was calculated to be −0.86 eV (vs. NHE), and that of PyAm‐PhAl‐COF was calculated to be −0.99 eV (vs. NHE) (Figure S10). Then the conduction band (CB) of PyAl‐PhAm‐COF is calculated to be −0.86 eV (vs. NHE), and the CB of PyAm‐PhAl‐COF is calculated to be −0.99 eV (vs. NHE) (Figure S10). These results were comparable to the band positions obtained from the Mott–Schottky test [46].

**FIGURE 4:**
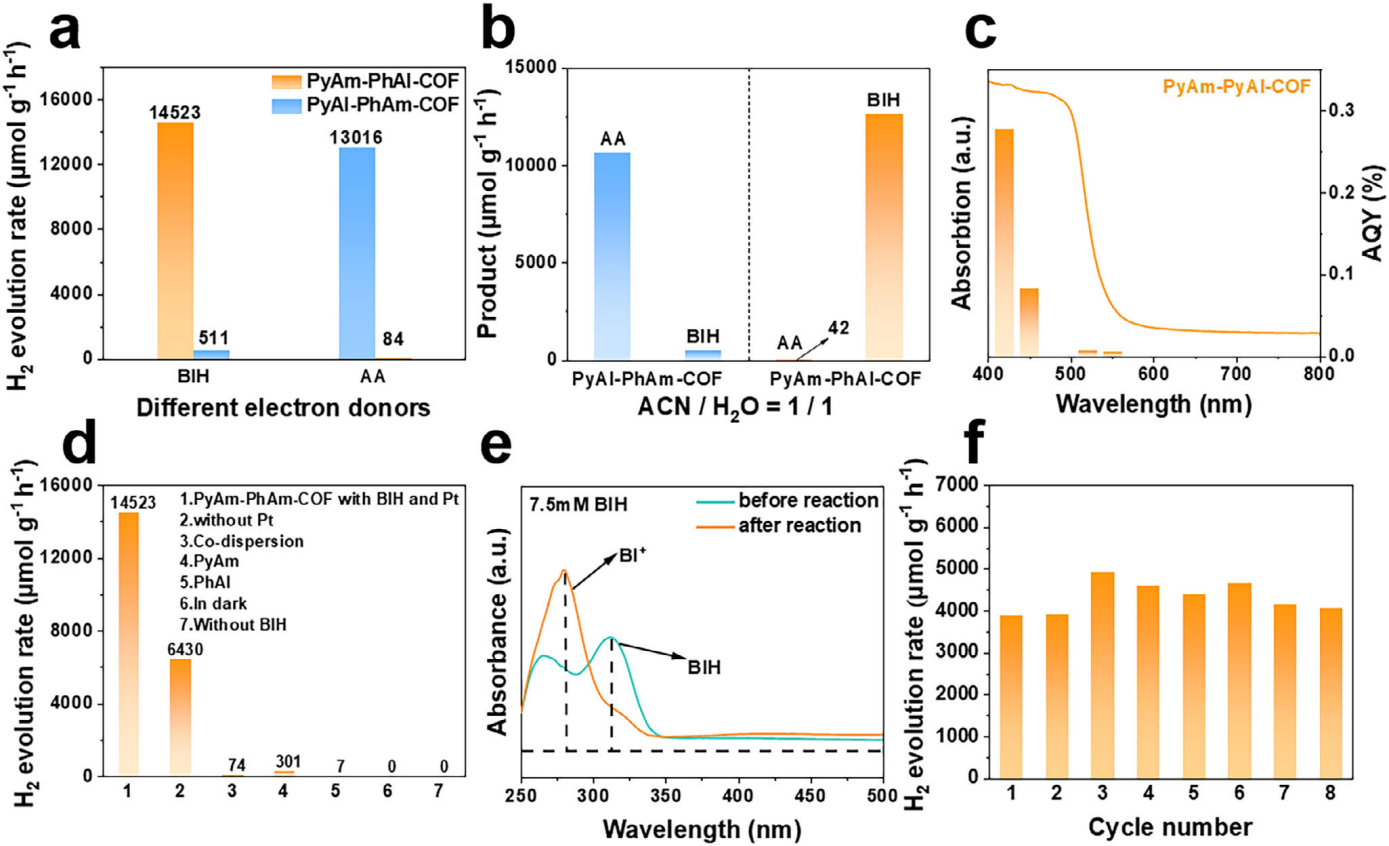
(a) Comparison of hydrogen evolution rates of two COFs with different electron donors. (b) Photocatalytic hydrogen evolution activity of two COFs in a uniform solvent environment. (c) Wavelength dependent AQY of photocatalytic H_2_ evolution for PyAm‐PhAl‐COF. (d) Control experiment results under different conditions. (e) UV–visible absorption spectra of BIH‐containing reaction solutions before and after photocatalysis. (f) Long‐term recycling photocatalytic hydrogen evolution of PyAm‐PhAl‐COF.

Subsequently, two COF isomers with identical donor–acceptor compositions but opposite imine bond linkage orientations were applied for photocatalytic hydrogen evolution under visible‐light irradiation (λ ≥ 420 nm). 3 wt.% Pt was employed as the cocatalyst by photodeposition. Either 1,3‐dimethyl‐2‐phenyl‐2,3‐dihydro‐1H‐benzo[d]imidazole (BIH) or ascorbic acid (AA) was used as the electron donor. The pH of the reaction solution was ∼11 when BIH was contained, and ∼2.5 when AA was contained. PyAm‐PhAl‐COF showed a high hydrogen evolution rate of 14 523 µmol g^−1^ h^−1^ in the presence of BIH, and this was approximately 170 times that observed with AA (84 µmol g^−1^ h^−1^) (Figure 4a; Figure S11). In contrast, PyAl‐PhAm‐COF performed poorly in the BIH system (511 µmol g^−1^ h^−1^), a trend consistent with previously reported D–A COFs having analogous imine orientations. When AA was used as the electron donor, PyAl‐PhAm‐COF achieved a rate of 13 016 µmol g^−1^ h^−1^, about 25 times higher than that observed in the BIH system. To exclude potential solvent effects on donor selectivity, control experiments conducted in a uniform solvent environment reproduced the same performance trend (Figure 4b), confirming that the photocatalytic behavior stems from intrinsic structural differences. These differences refer specifically to the molecular and electronic structures dictated by the orientation of the imine bonds, which fundamentally governs the driving force for charge separation. Notably, PyAm‐PhAl‐COF in the D─N═C─A form delivered one of the higher HER activities among reported D─N═C─A type COFs (Table S2). To quantify the photocatalytic activity of PyAm‐PhAl‐COF over the spectral distribution, the apparent quantum yield (AQY) for hydrogen evolution was measured. Measurements were carried out using band‐pass filters with center wavelengths of 420, 450, 520, and 550 nm, and the highest AQY obtained for PyAm‐PhAl‐COF was 0.27% at 420 nm (Figure 4c). Apparently, the AQY matches well with its absorption spectrum.

Furthermore, the PyAm‐PhAl‐COF based photocatalytic system using BIH as the sacrificial agent was investigated in depth. The control experiments were first conducted to investigate the contribution of each component to the HER reaction (Figure 4d). Bare PyAm‐PhAl‐COF (without Pt) also produced hydrogen in the presence of BIH under irradiation. The single monomers (PyAm and PhAl) as well as their physical mixture exhibited almost no activity, indicating that the covalently linked COF structure is vital for photocatalysis. Both light irradiation and the electron donor BIH were also indispensable for the photocatalytic HER. Moreover, various reaction conditions, including BIH concentration and solvent composition, were evaluated (Figure S12). Through systematic optimization of reaction conditions in photocatalytic reactions, the optimal reaction system was determined: hydrogen evolution activity peaked at a BIH concentration of 7.5 mm using an appropriate H_2_O/ACN mixed solvent ratio. Excessively high BIH concentrations inhibited the reaction due to light shielding or pore blockage, whereas imbalanced solvent ratios adversely affected either BIH solubility or proton concentration, thereby reducing hydrogen production efficiency. After hydrogen evolution reaction, BIH was oxidized to BI+, as verified by UV–vis absorption and mass spectrometry (Figure 4e; Figure S13) [47, 48]. Time‐dependent in situ diffuse reflectance infrared fourier transform (DRIFT) spectra similarly confirmed the progressive oxidation of BIH on the PyAm‐PhAl‐COF surface (Figure S14). In addition, AFM measurements revealed that the thickness of the COF particles gradually decreased from ∼120 to ∼20 nm over the reaction time (Figure S15). This thinning is possibly due to the exfoliation of the layered structure, which could lead to increased exposure of active sites and thus enhance the photocatalytic performance. In a long‐term recycling experiment, the hydrogen evolution rate gradually decreased as BIH was consumed, and recovered to a high level upon the addition of fresh BIH (Figure 4f). PyAm‐PhAl‐COF maintained a relatively stable hydrogen production rate over eight cycles, indicating good cycling stability. Post‐reaction characterization revealed attenuated XRD peaks and significantly reduced layer thickness by AFM, collectively indicating structural exfoliation during catalysis. The presence of lattice fringes and the uniform dispersion of Pt nanoparticles confirmed the structural integrity of the material after exfoliation, thus supporting its observed sustained catalytic performance (Figure S16) [49, 50].

The aforementioned experimental results clearly demonstrated that BIH exhibited exceptional photocatalytic hydrogen evolution performance in our COF system, with its activity far surpassing that of conventional sacrificial agents such as TEOA, TEA, and Na_2_S/Na_2_SO_3_ (Figure S17). Through comparative analysis, the fundamental reason for BIH's superior performance became evident. Thermodynamically, the most negative oxidation potential of BIH (0.56 V vs. NHE) provided the strongest driving force for hole capture (Figure S18a and Table S3) [51, 52, 53, 54, 55]. Regarding interfacial interactions, BIH achieved stable molecular pre‐assembly via strong π‐π stacking, with adsorption energies of −16.92 kcal/mol (PyAl‐PhAm‐COF) and −17.41 kcal/mol (PyAm‐PhAl‐COF). In contrast, TEOA and TEA relied on weaker C─H···π interactions, exhibiting lower adsorption energies: TEOA: −12.84 and −15.01 kcal/mol; TEA: −11.91 and −11.80 kcal/mol for the respective COF isomers [56]. The anions in Na_2_S/Na_2_SO_3_ suffered from significant electrostatic repulsion with the negatively charged COF surface (Figures S18b,S19,S20) [57, 58]. Most critically, in reaction kinetics, BIH's direct and rapid deprotonation enabled consecutive two‐electron transfer while avoiding recombination. This contrasted sharply with the slow intermolecular deprotonation of TEOA/TEA and the electron‐consuming pseudo‐cycle and light‐shielding effects inherent to Na_2_S/Na_2_SO_3_ [59, 60, 61]. The oxidation mechanism of BIH that enabled this advantage could be succinctly described as follows: BIH molecules pre‐assembled onto the hole‐rich pyrene units of the COF via *π*–*π* stacking. Upon photoexcitation, holes concentrated on these units were instantaneously captured by adjacent BIH, forming BIH^•+^. This radical cation rapidly deprotonated under alkaline conditions to generate the key BI^•^ radical. Acting as a potent reductant, it subsequently injected a second electron into the COF's conduction band. This unique “two‐electron, one‐proton” pathway ensured efficient hole scavenging and consecutive electron injection (Figure S21) [51]. In summary, the comprehensive superiority of BIH across thermodynamics, interfacial interaction, and reaction kinetics made it a suitable sacrificial agent in this COF‐based photocatalytic system.

Following the conclusion that BIH was the optimal sacrificial agent in this system, we further investigated the underlying interfacial mechanism. The results demonstrated that the surface charge of the COF materials varied systematically with pH. The zeta potential analysis revealed that under acidic conditions, both COFs showed positive values, indicating a positively charged surface; under alkaline conditions, they showed negative values, corresponding to a negatively charged surface (Figures S22 and S23) [62]. Specifically, under the optimal reaction conditions at different pH levels, the surface charge state effectively facilitated the adsorption of the corresponding sacrificial agents: the negatively charged ascorbic acid (existing predominantly as HA^−^) under acidic conditions, and the positively charged BIH radical cation (BIH^•+^) after oxidation under alkaline conditions (Figure S24) [51, 63]. This targeted adsorption was a key factor in enabling efficient charge separation and interfacial reaction kinetics.

**FIGURE 5:**
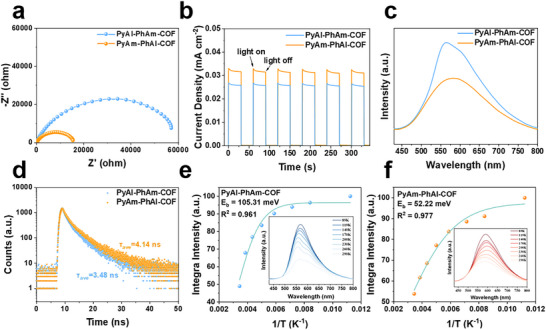
(a) Electro‐chemical impedance spectroscopy (EIS) Nyquist plots of PyAl‐PhAm‐COF and PyAm‐PhAl‐COF. (b) Transient photocurrent responses of PyAl‐PhAm‐COF and PyAm‐PhAl‐COF under irradiation with visible light. (c) Steady‐state photoluminescence spectra of PyAl‐PhAm‐COF and PyAm‐PhAl‐COF. (d) Time‐resolved photoluminescence spectra of PyAl‐PhAm‐COF and PyAm‐PhAl‐COF. (e) Exciton binding energies of PyAl‐PhAm‐COF, insert: temperature‐dependent PL spectra with excitation wavelength at 400 nm. (f) Exciton binding energies of PyAm‐PhAl‐COF, insert: temperature‐dependent PL spectra with excitation wavelength at 400 nm.

To gain deeper insight into the mechanisms behind the distinct photocatalytic behaviors of these two COFs, we investigated the corresponding photochemical properties and the charge carrier behaviors. Compared with PyAl‐PhAm‐COF, PyAm‐PhAl‐COF exhibited smaller electrochemical impedance, higher photocurrent density, and a stronger electron paramagnetic resonance (EPR) signal, indicating a more efficient charge separation and enhanced charge carrier mobility (Figure 5a,b; Figure S25). Steady‐state and time‐resolved photoluminescence (PL) spectroscopy were used to investigate the electron transfer capabilities of the two COFs. Steady‐state photoluminescence spectroscopy revealed that PyAl‐PhAm‐COF exhibited strong fluorescence emission, while PyAm‐PhAl‐COF showed significant fluorescence quenching, indicating that PyAm‐PhAl‐COF possessed more efficient electron transfer capabilities (Figure 5c). Further, time‐resolved photoluminescence spectroscopy analysis demonstrated that the average fluorescence lifetime of PyAm‐PhAl‐COF (4.14 ns) was longer than that of PyAl‐PhAm‐COF (3.48 ns) (Figure 5d; Figure S26), confirming that PyAm‐PhAl‐COF effectively suppressed the recombination of photogenerated electron‐hole pairs, thereby significantly enhancing its electron transfer efficiency. The properties of excitons were further investigated through temperature‐dependent fluorescence spectroscopy. The results revealed that the exciton binding energy of PyAm‐PhAl‐COF was approximately 52.22 meV, relatively lower than that of PyAl‐PhAm‐COF (105.31 meV) (Figure 5e,f). This difference indicates that excitons in PyAm‐PhAl‐COF are more prone to dissociate into free charge carriers, thereby facilitating the separation and migration of electrons and holes. These findings are consistent with the aforementioned fluorescence quenching and extended lifetime, further confirming the superior electron transfer performance of PyAm‐PhAl‐COF. The characterization conducted under neutral conditions demonstrates that PyAm‐PhAl‐COF exhibits more efficient photo electron transport than PyAl‐PhAm‐COF. Together, this enhanced kinetics and the more negative conduction band potential constitute the core of its performance advantage, a direct consequence of their distinct intrinsic structures.

**FIGURE 6:**
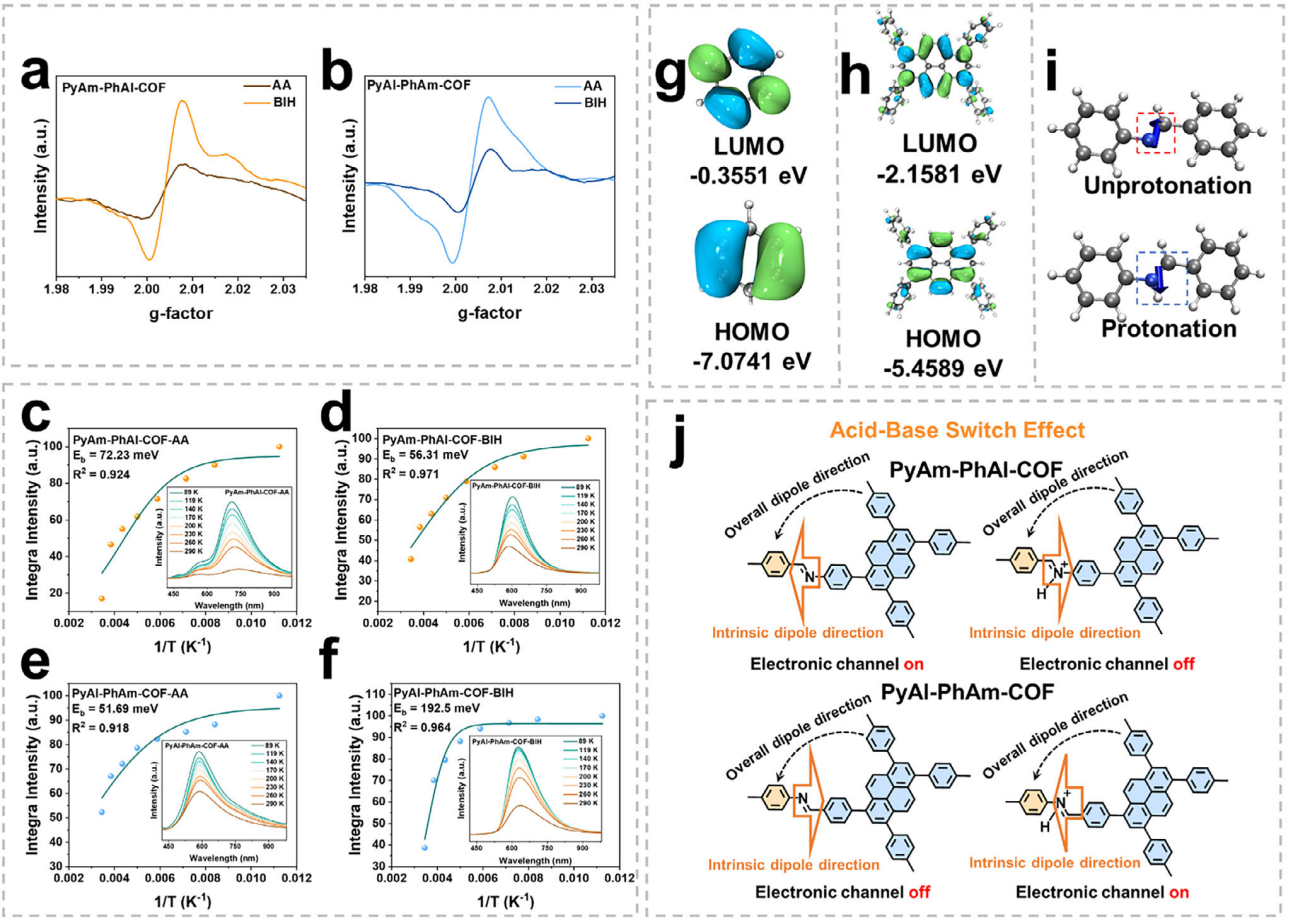
Comparison of EPR of (a) PyAm‐PhAl‐COF and (b) PyAl‐PhAm‐COF under different sacrificial agents. (c–f) Exciton binding energies of PyAm‐PhAl‐COF and PyAl‐PhAm‐COF under AA and BIH conditions, respectively; insets show temperature‐dependent fluorescence spectra measured at an excitation wavelength of 400 nm under the corresponding conditions. The HOMO/LUMO of (g) benzene unit and (h) pyrene unit. (i) Intrinsic dipole direction before and after COF protonation. (j) Mechanism diagram of “acid‐base switch effect”.

Subsequent EPR and temperature‐dependent PL studies on solid‐state COFs treated with AA or BIH were performed to investigate the pH‐responsive charge separation dynamics. EPR studies showed significant differences in the interactions of COFs with two electron donors. Only weak EPR signals were detected for the COF‐AA and COF‐BIH systems in the dark, whereas the signal intensity was enhanced after visible light irradiation, confirming an efficient photo‐induced electron transfer process (Figure S27). PyAm‐PhAl‐COF‐BIH exhibited a stronger EPR signal intensity than PyAm‐PhAl‐COF‐AA (Figure 6a), confirming the selective preference of PyAm‐PhAl‐COF for BIH. In contrast, the significantly enhanced signal of PyAl‐PhAm‐COF‐AA compared to PyAl‐PhAm‐COF‐BIH revealed its preferential synergy with AA (Figure 6b). The temperature‐dependent fluorescence spectra further supported these findings. PyAm‐PhAl‐COF‐BIH (56.31 meV) exhibited a smaller exciton binding energy than PyAm‐PhAl‐COF‐AA (72.23 meV) (Figure 6c,d). This decrease indicates that the interaction between PyAm‐PhAl‐COF and BIH facilitated exciton dissociation into free charge carriers, thereby promoting the separation and migration of electrons and holes. In contrast, PyAl‐PhAm‐COF displayed the opposite behavior. PyAl‐PhAm‐COF‐AA exhibited a smaller exciton binding energy (51.69 meV) compared to PyAl‐PhAm‐COF‐BIH (192.5 meV) (Figure 6e,f). This suggests that the interaction between PyAl‐PhAm‐COF and AA was more favorable for exciton dissociation.

To elucidate the regulatory mechanism by which imine bond orientation reverses the photocatalytic activity of COFs in acidic/basic environments, we conducted density functional theory (DFT) calculations. First, we computationally determined the HOMO and LUMO orbitals of the pyrene and benzene ring monomers constituting the COF, thereby clarifying its donor‐acceptor (D–A) structure. The results revealed that the HOMO energy level of the pyrene unit was higher than that of the benzene ring unit, indicating stronger electron delocalization and greater favorability for charge transfer (Figure 6g,h). Furthermore, pyrene consists of four fused benzene rings, resulting in highly delocalized electrons throughout the molecular framework, which further promotes charge migration. Therefore, during photocatalysis, pyrene acts as an electron donor while the benzene rings serve as electron acceptors. Subsequently, the intrinsic dipole directions on the imine bond unit under acidic and basic conditions were calculated. As shown in Figure 6i, when the COF is unprotonated in a basic environment, the intrinsic dipole direction of the imine bond points from the N atom toward the C atom. After protonation in an acidic environment, the dipole direction reverses to point toward the C atom adjacent to N. This structural‐level dynamic reversal behavior leads to a typical “molecular diode effect” (Figure 6j), which constitutes the microscopic mechanism underlying the material's pH‐responsive rectifying properties. Specifically, in PyAm‐PhAl‐COF, the dipole orientation of the imine bond in the unprotonated state (N pointing toward C) aligns with the material's overall dipole direction, forming a “forward diode” channel that facilitates electron transfer driven by the basic sacrificial agent BIH. Upon protonation, the dipole reverses to C pointing toward N, opposing the overall dipole and blocking electron transport. Conversely, in PyAl‐PhAm‐COF, the dipole of the imine bond in the unprotonated state (N pointing toward C) opposes the overall dipole direction, maintaining a “reverse diode” blocking state. Protonation induced by the acidic sacrificial agent AA flips the dipole to C pointing toward N, reestablishing a channel aligned with the overall electric field and thereby enabling electron transport. This “molecular diode effect” fundamentally reveals that the imine bond, acting as a pH‐responsive unit, can dynamically regulate electron transport pathways and intelligently switch photocatalytic activity through chemically selective reversal of the dipole orientation.

## 3 Conclusion

This work demonstrates the construction of two imine‐linked COF photocatalysts guided by a molecular diode design strategy. Through precise control of donor‐acceptor bonding orientation, these materials exhibit remarkable pH‐responsive rectification characteristics during photo‐induced electron transfer. PyAm‐PhAl‐COF exhibits exceptional hydrogen evolution performance under alkaline conditions, achieving an activity 172 times higher than that in an acidic environment. In contrast, PyAl‐PhAm‐COF shows an inverse trend, with a 25‐fold enhancement in hydrogen production under acidic compared to alkaline conditions. This distinctive pH‐responsive behavior stems from protonation/deprotonation‐induced dynamic reversal of molecular dipoles, which effectively modulates the accessibility of electron transport channels. Our work establishes a new mechanism for regulating charge separation and transfer pathways in COFs via molecular‐level structural engineering and offers a designable strategy for developing stimuli‐responsive smart photocatalytic systems.

## Conflicts of Interest

The authors declare no conflicts of interest.

## Supporting information




**Supporting File**: advs73692‐sup‐0001‐SuppMat.docx.

## Data Availability

The data that support the findings of this study are available from the corresponding author upon reasonable request.
