# Supplementary material for: Molecular Diode‐Based Covalent Organic Frameworks: Imine Orientation‐Driven Acid‐Base Switching Photocatalytic H2 Production
Source: Adv Sci (Weinh). 2026 Jan 5;13(16):e22414. doi: 10.1002/advs.202522414 (PMC13042519; doi:10.1002/advs.202522414)
Supplement: Supplementary file 1 — Supporting File: advs73692‐sup‐0001‐SuppMat.docx. [file ADVS-13-e22414-s001.docx]

**Supplementary Information**

**Molecular Diode-Based Covalent Organic Frameworks: Imine Orientation-Driven Acid-Base Switching Photocatalytic H_2_ Production**

Tianyi Liu^a^, Yunjie Lang^a^, Ning Sun^a^, Xu Fang^a^, Jinhua Rao^b^, Yuchun Xu^a^, Zhen Li^*a^, Weiqiao Deng^*a^

^a^ Institute of Frontier Chemistry, School of Chemistry and Chemical Engineering, Shandong University, No.72 Binhai Road, Qingdao, Shandong, 266237, China

^b^ School of Integrated Circuits, Ludong University, Yantai, Shandong, 264025, China

Corresponding authors:

E-mail:zhen_li@sdu.edu.cn and dengwq@sdu.edu.cn

**Section I: Supplementary methods**

- **S1 Materials and methods**

All chemicals were purchased from commercial reagent companies without further treatment. n-Butanol (n-BuOH), o-dichlorobenzene (o-DCB), tetrahydrofuran, acetonitrile and aqueous acetic were bought from Sinopharm Chemical Reagent Co., Ltd. 4,4',4'',4'''-(pyrene-1,3,6,8-tetrayl) tetraaniline (PyAm), terephthalaldehyde (PhAl), 4,4,4,4-(Pyrene-1,3,6,8-tetrayl)tetrabenzaldehyde (PyAl), p-Phenylenediamine (PhAm) were bought from Jilin Chinese Academy of Sciences-Yanshen Technology. 1,3-Dimethyl-1,3-dihydro-2-phenyl-2H-benzimidazole (BIH) and ascorbic acid (AA) were bought from Shanghai BiDe Pharmaceutical Technology Co.

The Brunauer-Emmett-Teller (BET) surface areas of COFs were measured at 77 K by using a Quantachrome Automated Surface Area & Pore Size Analyzer. Pore size distributions was estimated by nonlocal density functional theory (NLDFT). The powder X-ray diffraction (PXRD) pattern was recorded on a Cu-Kα X-ray radiation source (λ=0.154056 nm) incident radiation by a Rigaku MiniFlEX 600 instrument over the range of 2θ =2.0~40.0° with a step size of 0.02° per step. The FT-IR spectra were recorded by Thermo Nicolet iS50 in the range from 400 to 4000 cm-1. Solid-state ^13^C CP/MAS NMR spectra were recorded on 400WB S2 AVANCE III (Bruker, Switzerland) plus 400 MHz spectrophotometer at 298 K. Morphological information for COFs were obtained from field-emission scanning electron microscope (SEM, FEI Nano 450), transmission electron microscopy (TEM, JEOL jem 2100f) and high-angle annular dark-field scanning transmission election microscope (HAADF-STEM, FEI Themis Z).Thermogravimetric analyses (TGA) was recorded on a Netzsch Model STA 449C microanalyzer heated from 25 °C to 900 °C in nitrogen atmosphere. UV-visible absorption spectra of the polymers were measured on a Shimadzu UV-2550 UV-vis spectrometer by measuring the reflectance of powders in the solid state. High-resolution valence band ultraviolet photoelectron spectra (UPS) were obtained from Thermo Fisher Scientific Escalab 250Xi. Fluorescence spectrum and fluorescence lifetime were measured using a FLS1000 Edinburgh Instruments spectrofluorimeter. The Photocatalytic hydrogen evolution measurements were carried out in a Pyrex top-irradiation reaction vessel connected to a glass closed Labsolar 6A gas circulation system (Perfect Light) and gas products were analyzed by online 8890 GC System (Agilent) referencing against standard gas with a known concentration of hydrogen. The light intensities of high precision illuminator system LX300f were tested by the PM100D optical power meter (Tech Support). The electrochemical measurements were recorded on the NOVA II electrochemical workstation with a standard three-electrode system with the photocatalyst-coated FTO as the working electrode, Pt plate as the counter electrode and the Ag/AgCl electrode as a reference electrode. The water vapor adsorption and desorption curves were tested at German MICROTRAC MRB Belsorp max II.

- **S2 Photocatalytic hydrogen evolution measurement**

The photocatalytic hydrogen evolution measurements were carried out in a Pyrex top irradiation reaction vessel connected to a glass closed gas circulation system (Labsolar 6A, Perfect Light). For PyAm-PhAl-COF reaction, it was conducted using 10 mg photocatalyst, 50 mL H_2_O ,50mL ACN and 168 mg BIH as sacrificial agent, and the mixture was dispersed by ultrasonication for 30 minutes to obtain a uniform dispersion. After that, appropriate H_2_PtCl_6_ aqueous solution (0.376 g Pt L^-1^) was added into the solution system. For PyAl-PhAm-COF reaction, it was conducted using 10 mg photocatalyst, 100 mL H_2_O and 850 mg ascorbic acid as sacrificial agent, and the mixture was dispersed by ultrasonication for 30 minutes to obtain a uniform dispersion. After that, appropriate H_2_PtCl_6_ aqueous solution (0.376 g Pt L^-1^) was added into the solution system. Then the samples were added into a quartz transparent photoreactor. The above suspension was bubbled with Argon for 30 minutes, and was kept at 25 °C using circulating water. The Xenon lamp was turned on to start the photocatalysis measurements. Besides, the reaction solution was constantly stirred to maintain the entire mixture homogeneous. Hydrogen dissolved in the reaction mixture was not measured and the pressure increase generated by the evolved hydrogen was neglected in the calculations. The hydrogen evolution rates were determined from a linear regression fit. After the photocatalysis experiment, the photocatalysts were recovered by washing with water then dried at 120 °C vacuum oven.

- **S3 Long-term photocatalytic experiment**

The long-term photocatalytic hydrogen evolution measurements were carried out in a same system as the photocatalytic hydrogen evolution measurements. It was conducted using 10 mg PyAm-PhAl-COF, 50 mL ACN, 50 mL H_2_O and 56 mg BIH as sacrificial agent, and the mixture was dispersed by ultrasonication for 30 minutes to obtain a uniform dispersion. After that, 3 wt% Pt (795 μL 0.376 g Pt L^-1^H_2_PtCl_6_ aqueous solution) was added into the solution system. During the long-term experiment, 56 mg BIH was added for 7 times only when the amount of hydrogen no longer increased.

- **S4 Apparent quantum yield (AQY) measurement**

The AQY measurement was conducted in the same reaction system as other photocatalytic reactions, otherwise the xenon lamp was equiped with a band-pass filter with central wavelength of 420 nm and full-width at half-maximum (FWHM) of ~10 nm. The number of photons reaching the solution was measured using a calibrated Si photodiode. For full absorption of the incident photons, 10 mg 3% COF was used as photocatalyst in the AQY measurement. The AQY value was calculated according to the following equation:

$$\text{AQY}\left( \text{\%} \right)\text{=}\frac{\text{2×Number of evolved }\text{H}_{\text{2}}\text{ molecules}}{\text{Number of indicent photos}}\text{×100\%}$$

$$\text{=}\frac{\text{2×M×}\text{N}_{\text{A}}}{\text{S×P×t×}\frac{\text{λ}}{\text{h×c}}}\text{×100\%}$$

Where M is the amount of H_2_ production amount (μmol) per hour, *N*_A_ is Avogadro constant (6.02 ×10^23^ mol^-1^), *h* is the Planck constant (6.626 × 10^-34^ J·s), *c* is the speed of light (3.0 × 10^8^ m s^-1^), *S* is the irradiation area 10 cm^2^, *P* is the intensity of irradiation light (W cm^-2^), *t* is the photoreaction time (s), *λ* is the wavelength of the monochromatic light (nm).

- **S5 Photoelectrochemical measurements**

There are two preparations before the test: Firstly, FTO glasses were firstly cleaned by sonication in ethanol for 30 min and dried under nitrogen flow. Secondly, the working electrodes were immersed in the electrolyte for 60 s before any measurements were taken. After that, the photocurrent-time (i-t) profiles, electrochemical impedance spectra (EIS), Mott-Schottky plot were recorded on the NOVA II electrochemical workstation with a standard three-electrode system with the photocatalyst-coated FTO as the working electrode, Pt plate as the counter electrode and the Ag/AgCl electrode as a reference electrode. A 0.2 M Na_2_SO_4_ solution (pH = 6.8) was used as the electrolyte. A 300 W Xenon lamp with a 420 nm cut-off filter was used as the light source during the measurement. The applied potentials vs. Ag/AgCl is converted to RHE potentials using the following equation:

- **S6 Fluorescence spectrum**

Steady-state PL, Temperature-dependent PL spectra and phosphorescence spectrum were acquired using Edinburgh Instruments, FLS980 spectrometer. The exciton binding energy could be calculated as follows:

$$\text{I}\left( \text{T} \right)\text{=}\frac{\text{I}_{\text{0}}}{\text{1+A}\text{e}^{\text{-}\text{E}_{\text{b}}\text{/}\text{k}_{\text{B}}\text{T}}}$$

- **S7 Calculations details**

All the calculations were performed using the Gaussian 16 software package ^[1]^ with DFT methods, incorporating the SMD ^[2]^ solvent model. Geometry optimizations employed the B3LYP hybrid functional ^[3-5]^ with D3 dispersion correction ^[6]^. Energy calculations utilized the 6-311+G (2d, p) basis set.

- **S8. Synthetic procedures**

**8.1 PyAl-PhAm-COF.** A n-butylalcohol (n-BuOH)/o-dichlorobenzene (o-DCB)/6M AcOH (5/5/1 by vol.; 2.2 mL) mixture of p-Phenylenediamine (0.096 mmol, 10.44 mg) and 4,4,4,4-(Pyrene-1,3,6,8-tetrayl)tetrabenzaldehyde (0.048 mmol, 30 mg) in a Pyrex tube (20 mL) was degassed by three freeze-pump-thaw cycles. The tube was sealed off and heated at 120 °C for 3 days. The precipitate was collected by centrifugation, and washed with anhydrous THF for 5 times. The powder was dried at 120 °C under vacuum overnight.


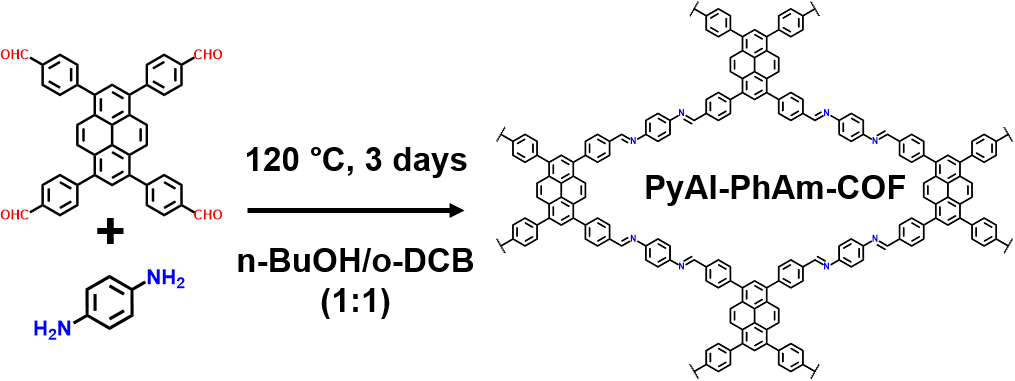


**Figure S1** Synthesis processes of PyAl-PhAm-COF

**8.2 PyAm-PhAl-COF.** A n-butylalcohol (n-BuOH)/o-dichlorobenzene (o-DCB)/6M AcOH (5/5/1 by vol.; 2.2 mL) mixture of terephthalaldehyde (0.16 mmol, 21.5 mg) and 4,4',4'',4'''-(pyrene-1,3,6,8-tetrayl) tetraaniline (0.08 mmol, 45.2 mg) in a Pyrex tube (20 mL) was degassed by three freeze-pump-thaw cycles. The tube was sealed off and heated at 120 °C for 3 days. The precipitate was collected by centrifugation, and washed with anhydrous THF for 5 times. The powder was dried at 120 °C under vacuum overnight.


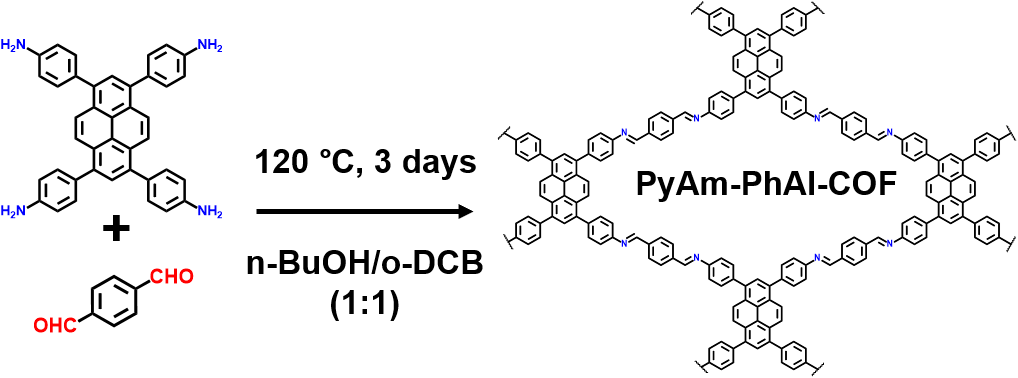


**Figure S2** Synthesis processes of PyAm-PhAl-COF

- **S9. Statistical Analysis.** Data are presented as mean values and were analyzed using OriginPro 2025. All experiments were performed in at least three independent replicates to ensure reproducibility. Hydrogen evolution rates were normalized to μmol g^-1^ h^-1^.

**Section II: Supplementary Figures and Tables.**


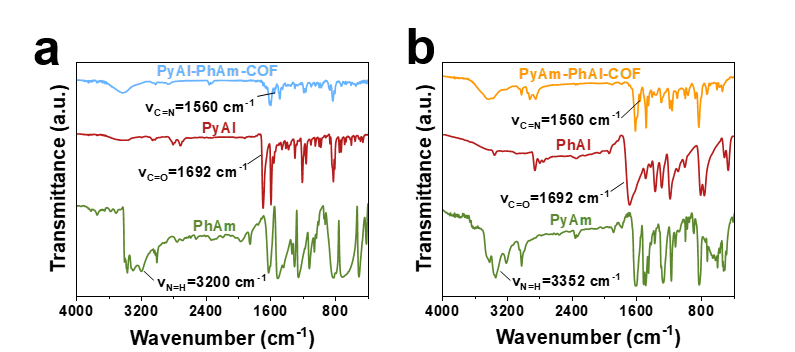


**Figure S3** Infrared spectra of (a) PyAl-PhAm-COF and (b) PyAm-PhAl-COF.


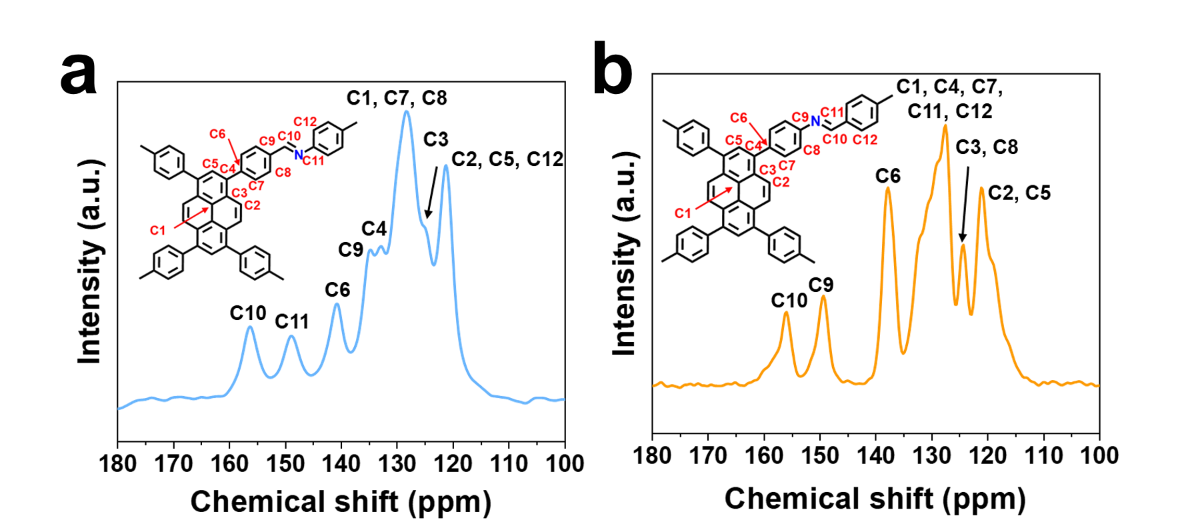


**Figure S4** Solid state ^13^C NMR spectrum of (a) PyAl-PhAm-COF and (b) PyAm-PhAl-COF.


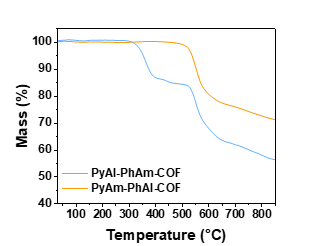


**Figure S5** TGA profile for PyAl-PhAm-COF and PyAm-PhAl-COF.


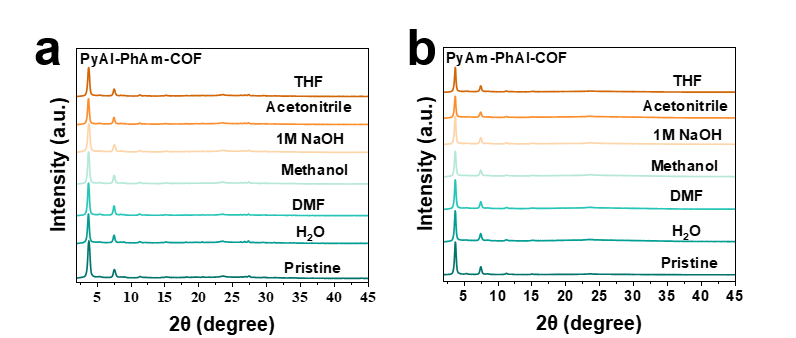


**Figure S6** PXRD patterns of the (a) PyAl-PhAm-COF and (b) PyAm-PhAl-COF after soaking in difference solvents for 4 hours.


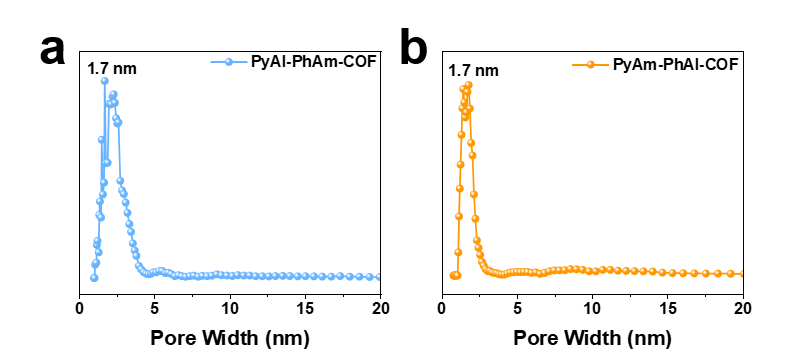


**Figure S7** Pore size distribution for (a) PyAl-PhAm-COF and (b) PyAm-PhAl-COF.


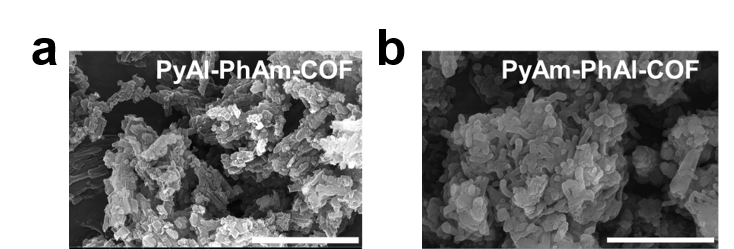


**Figure S****8** SEM image for (a) PyAl-PhAm-COF (scale bar,3 μm) and (b) PyAm-PhAl-COF (scale bar,5 μm).


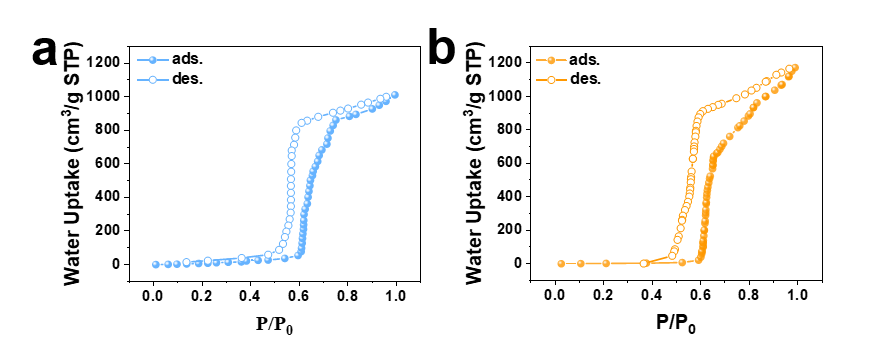


**Figure S9** Water adsorption and desorption isotherms of (a) PyAl-PhAm-COF and (b) PyAm-PhAl-COF.


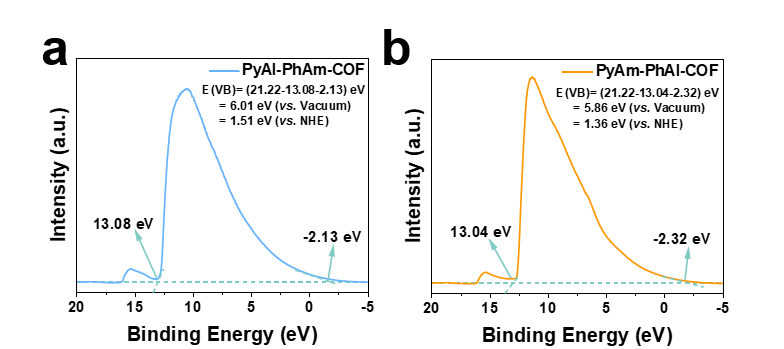


**Figure S10** UPS spectrum of (a) PyAl-PhAm-COF and (b) PyAm-PhAl-COF.


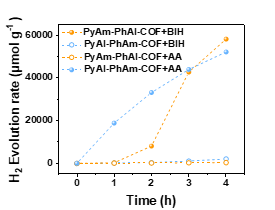


**Figure S11** Photocatalytic hydrogen evolution activity of two COFs under visible light irradiation.


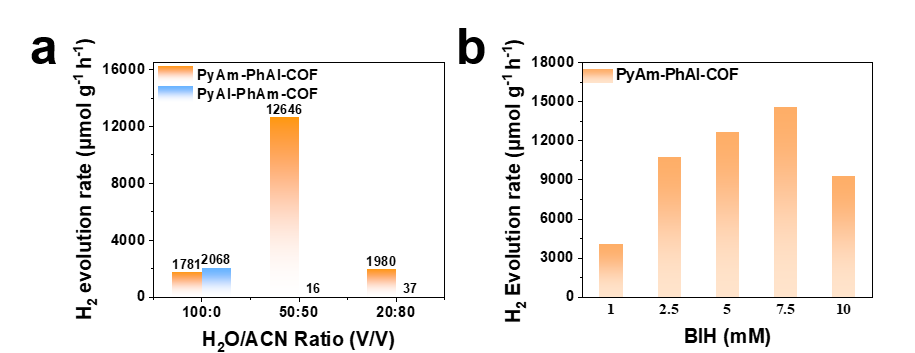


**Figure S12** Adjusting (a) the solvent system and (b) the concentration of BIH in the photocatalytic system.


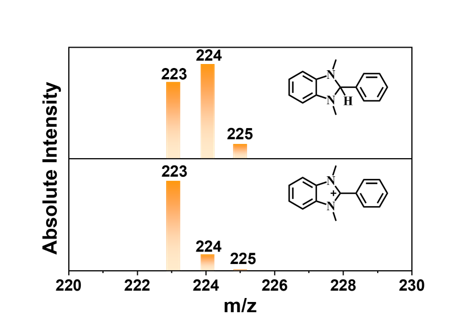


**Figure S13** GC-MS plots before and after BIH reaction.


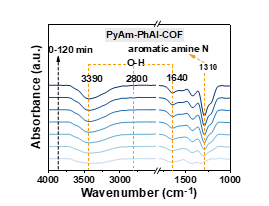


**Figure S14** In situ DRIFT measurement of PyAm-PhAl-COF/BIH based photocatalytic system.


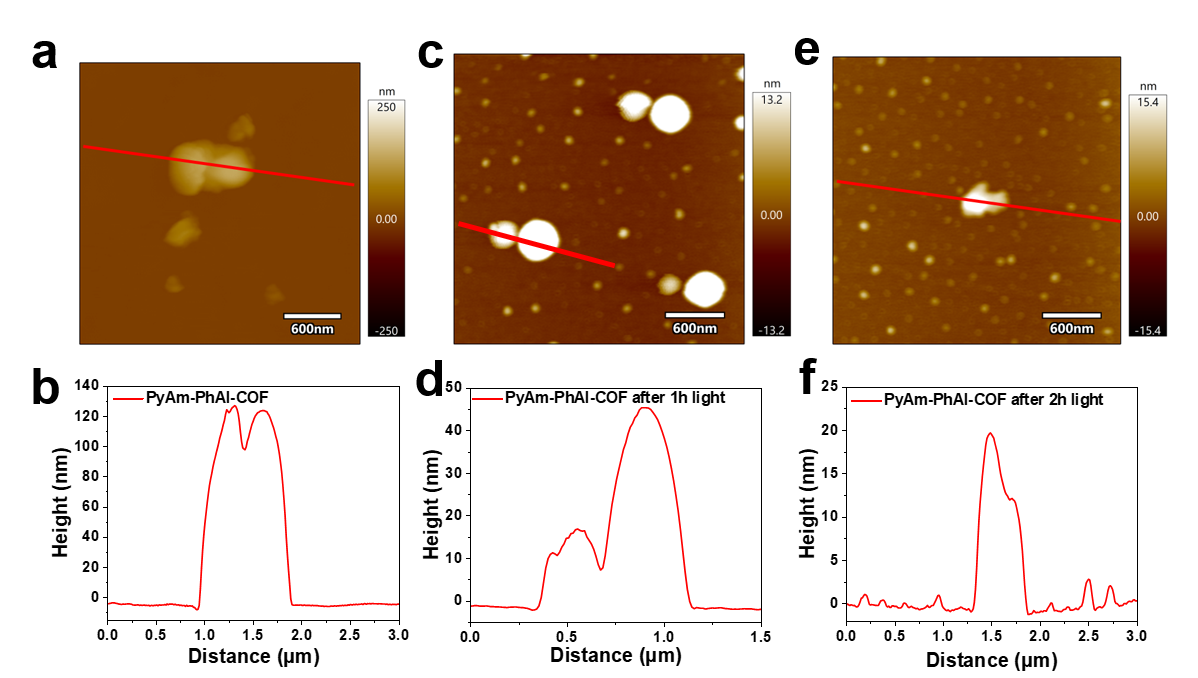


**Figure S15** AFM of PyAm-PhAl-COF (a) and (b) without illumination, (c) and (d) with illumination for 1 hour, and (e) and (f) with illumination for 2 hours.


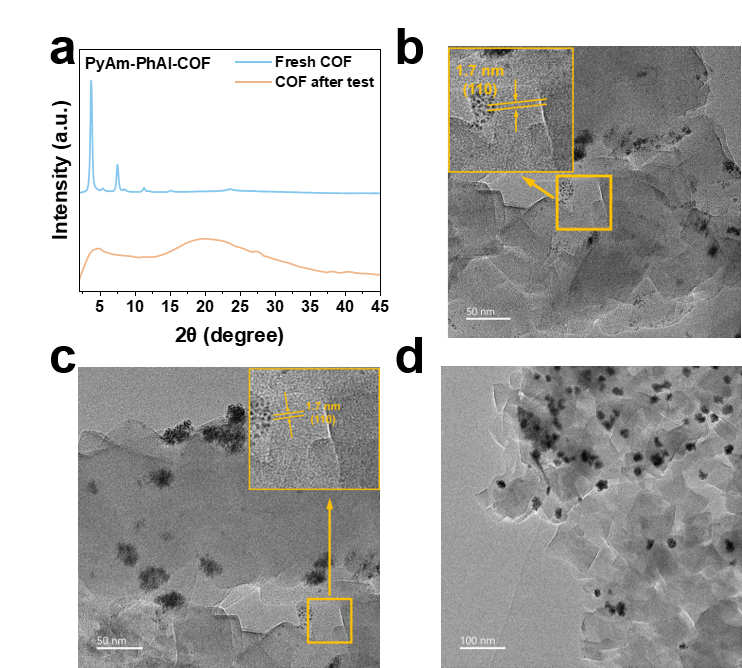


**Figure S16** Structural characterization of PyAm-PhAl-COF after long-term cycling tests. (a) XRD of the fresh and spent COF. (b, c, d) TEM images of the spent catalyst at different magnifications


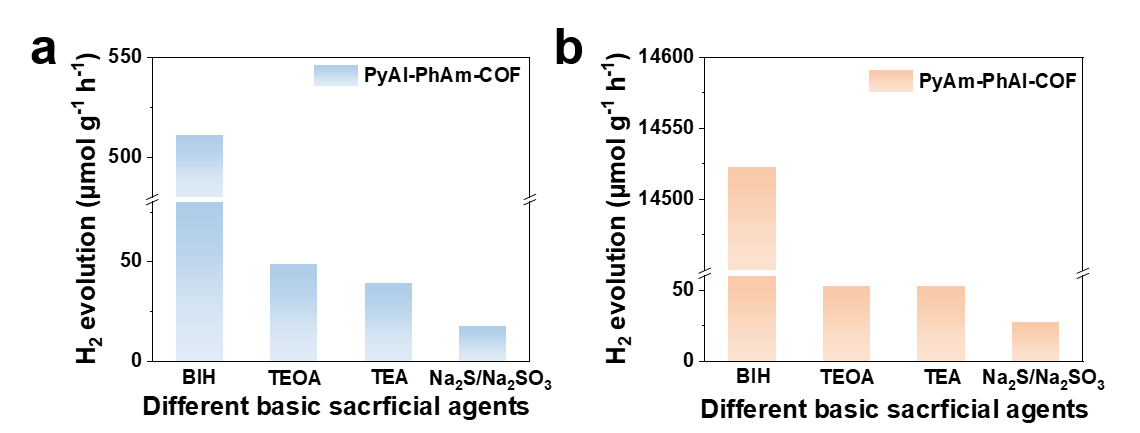


**Figure S17** Photocatalytic hydrogen production activity of COF and other base sacrificial agents


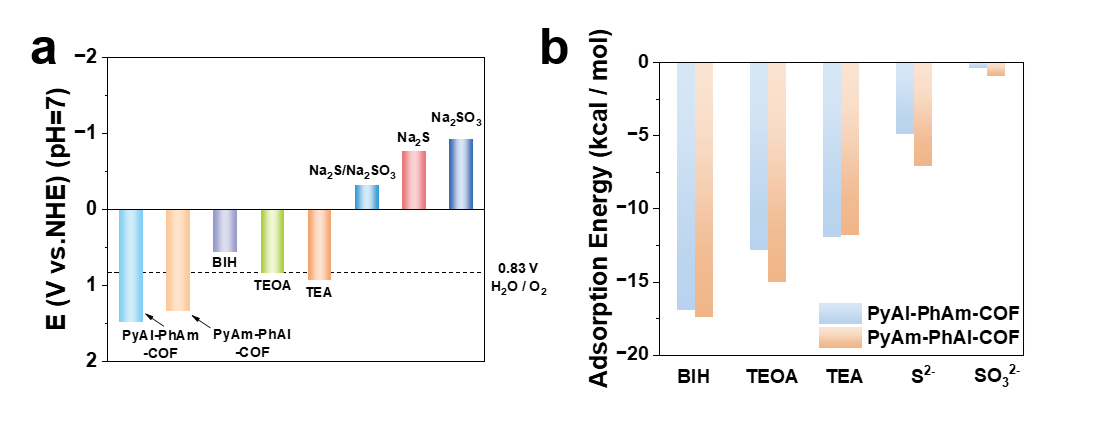


**Figure S18 (**a) Energy diagram for COF and different base sacrificial agent. (b) Adsorption energy of different base sacrificial agents and COFs.


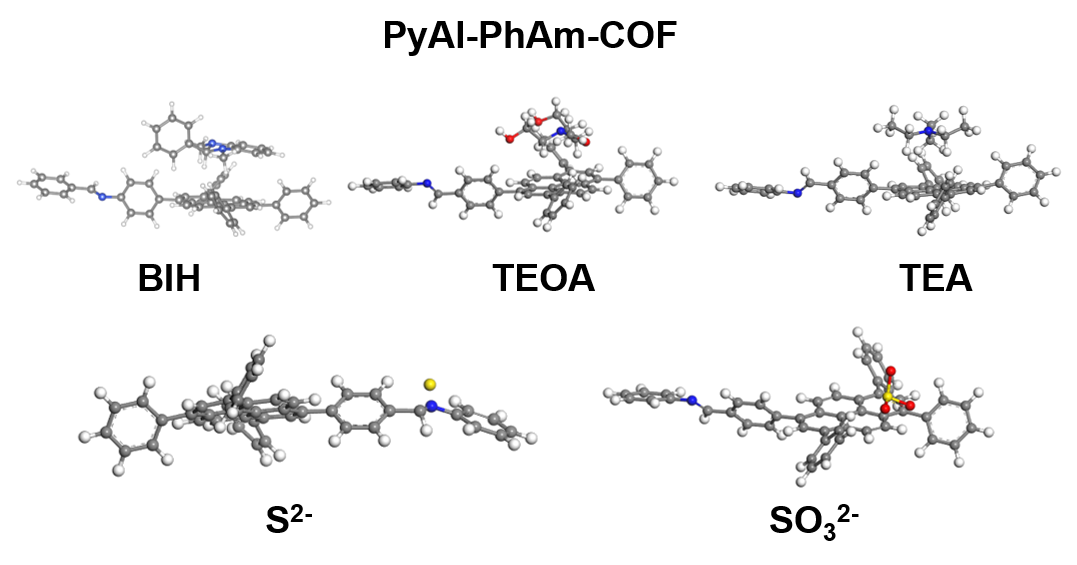


**Figure S19** Adsorption configurations of PyAl-PhAm-COF and different sacrificial agents.


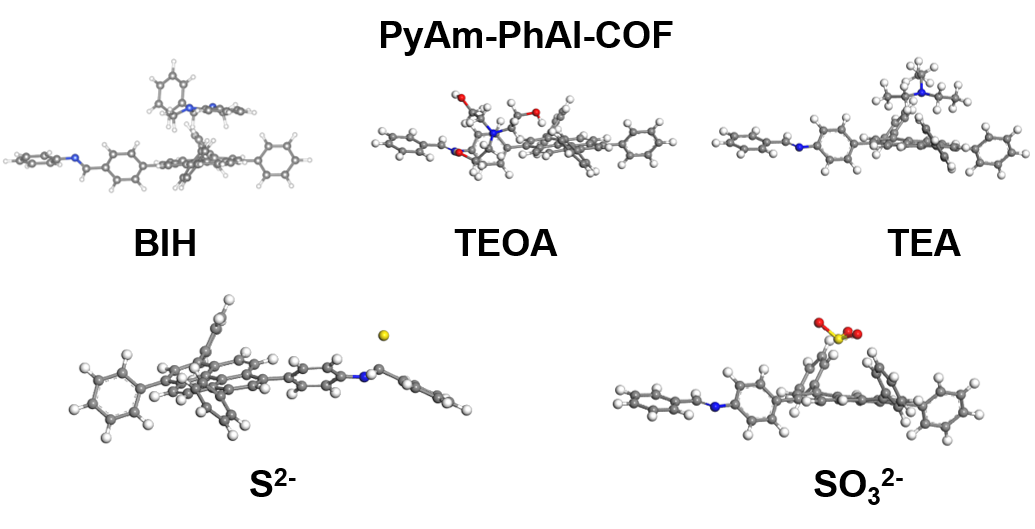


**Figure S20** Adsorption configurations of PyAm-PhAl-COF and different sacrificial agents.


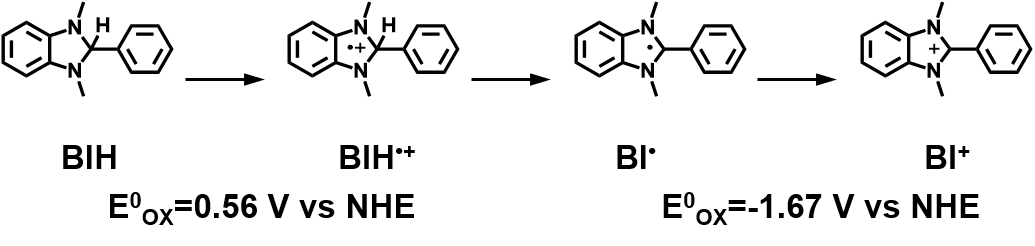


**Figure S21** Oxidation and deprotonation process of BIH.


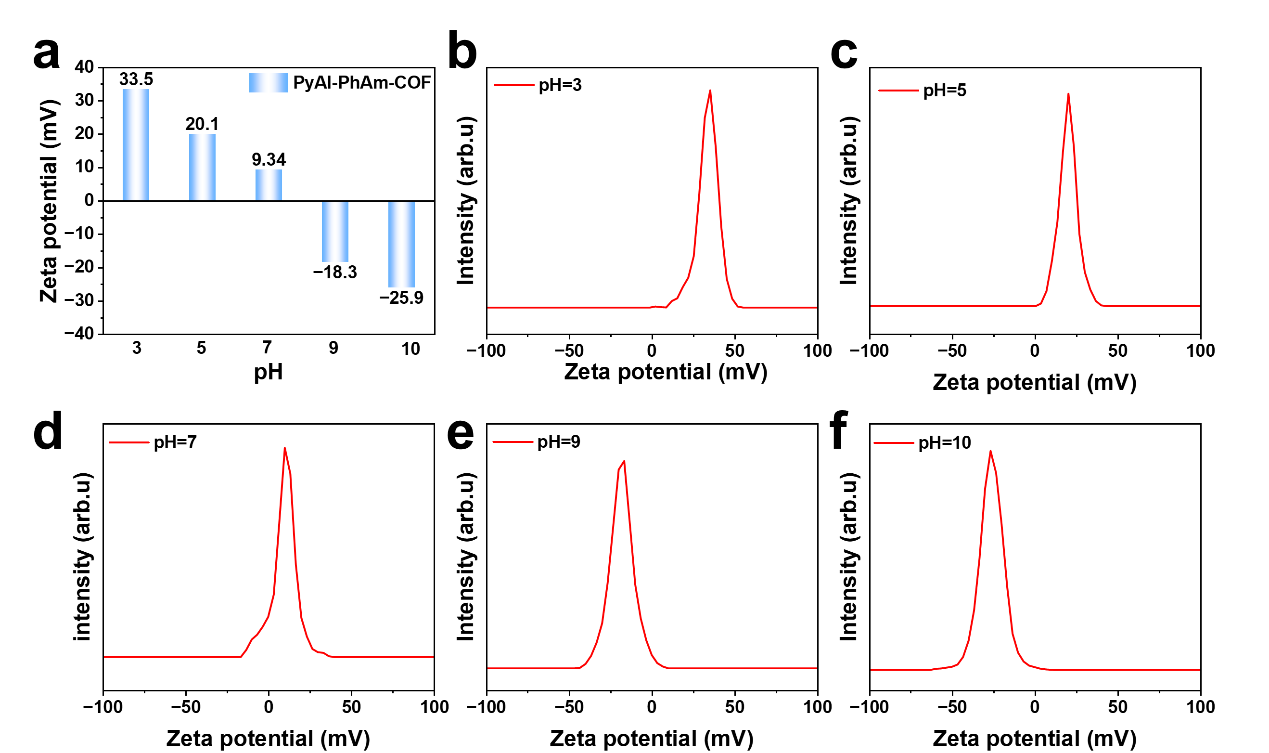


**Figure S22** Zeta potential of PyAl-PhAm-COF.


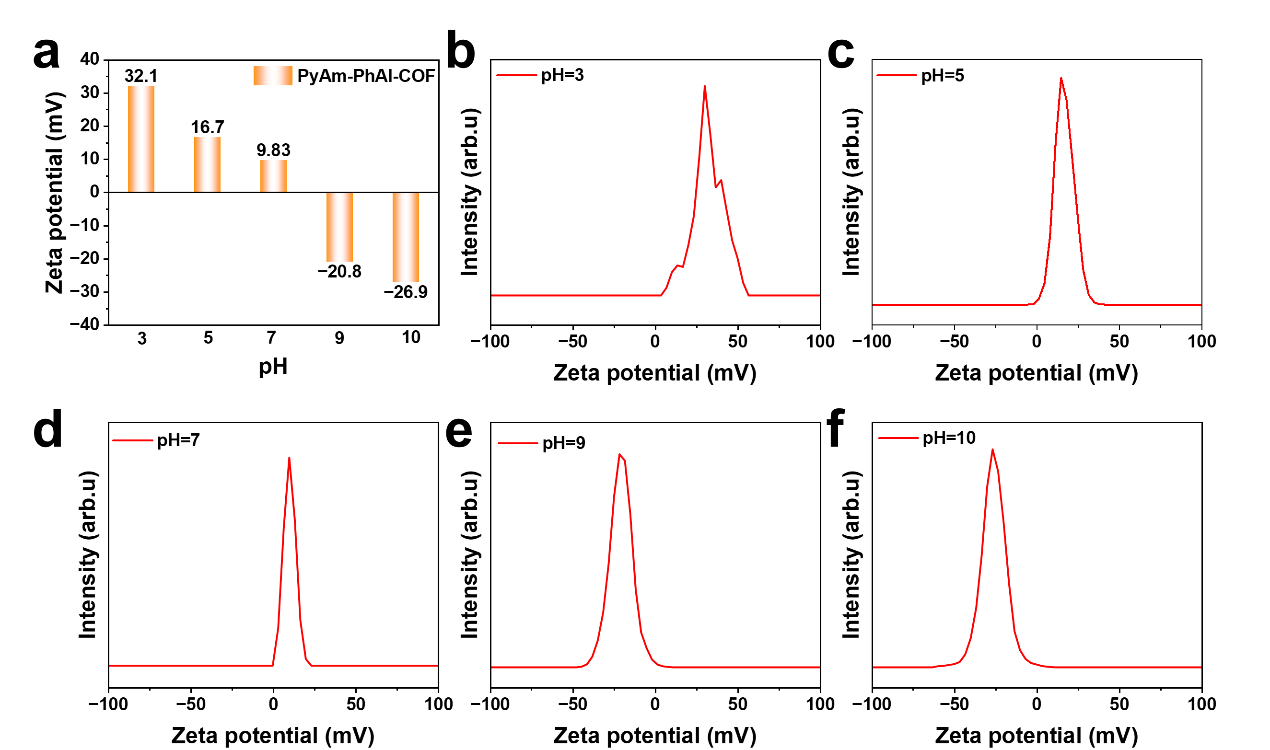


**Figure S23** Zeta potential of PyAm-PhAl-COF.


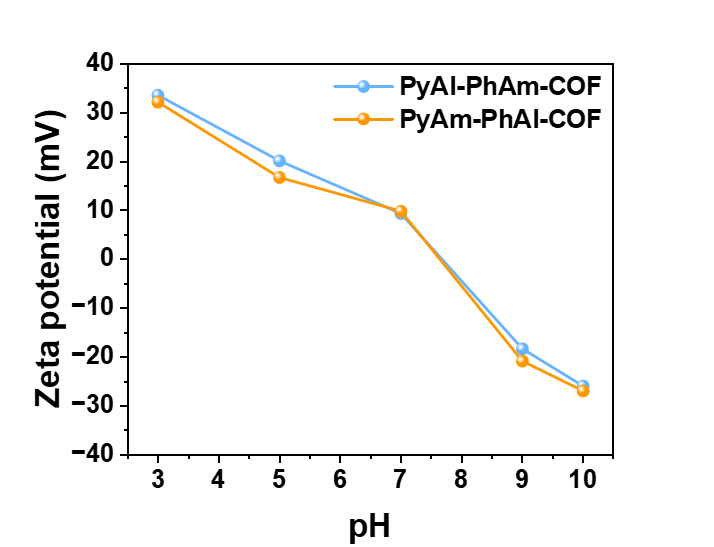


**Figure S24** Zeta potential of PyAl-PhAm-COF and PyAm-PhAl-COF.


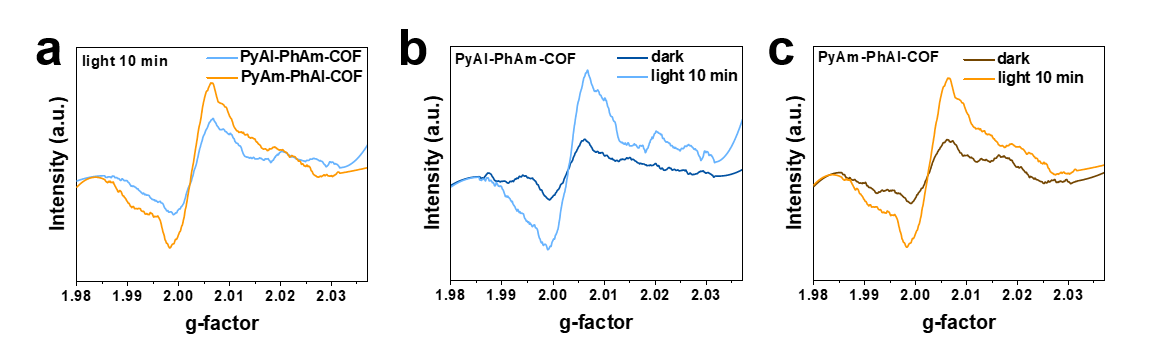


**Figure S25** (a) Comparison of ESR of two COFs. ESR of (b). PyAl-PhAm-COF and (c) PyAl-PhAm-COF under dark and light conditions.


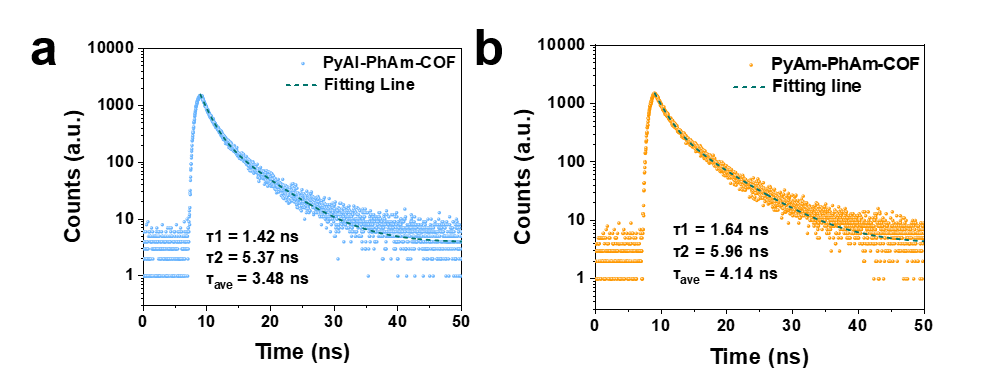


**Figure S26** Fluorescence lifetime decay of (a) PyAl-PhAm-COF and (b) PyAl-PhAm-COF.


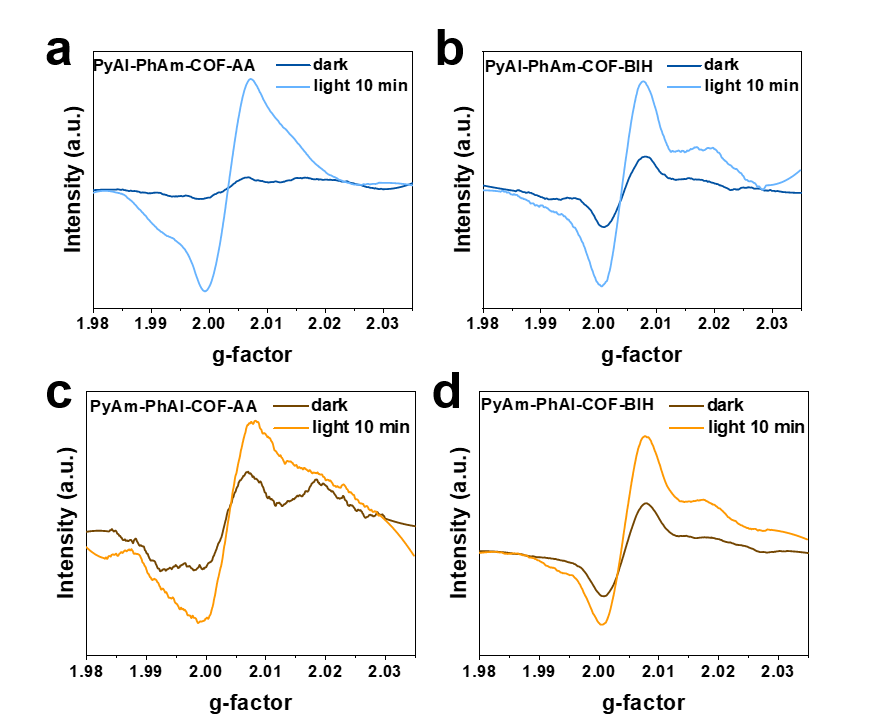


**Figure S27** EPR of COFs after protonation and deprotonation.

**Table S1** Pore size distribution of different COFs

| COF | Surface Area(m^2^g^-1^) | pore volume (cm^3^g^−1^) | pore width (nm) | Theoretically calculated pore size (Å) |
| --- | --- | --- | --- | --- |
| PyAl-PhAm-COF | 1100 | 0.967 | 1.7 | 18 |
| PyAm-PhAl-COF | 1162 | 1.233 | 1.7 | 17 |

**Table S2** Photocatalytic performance comparison of COF (DNCA) with other representative COF based photocatalysts.

| COF | Co-catalyst | SA | Illumination | Activity  (μmol g^-1^ h^-1^) | References |
| --- | --- | --- | --- | --- | --- |
| PyAm-PhAl-COF | Without Pt | BIH | λ>420 nm | 6430.69 | This work |
| PyAm-PhAl-COF | 3%Pt |  |  | 14523.7 |  |
| COF-921 | 3%Pt | AA | λ>420 nm | 8100 | [7] |
| COF-925 |  |  |  | 7900 |  |
| COF-932 |  |  |  | 79 |  |
| Cof-953 | 5%Pt | AA | λ>420 nm | 60535 | [8] |
| Py-N-DBT-COF | Without Pt | AA | λ>420 nm | 241.3 | [9] |
| Py-N-DBT-COF | 5%Pt |  |  | 828.6 |  |
| PyAm-TpbAl-COF | 3%Pt | AA | λ>420 nm | 700 | [10] |
| DNCA-1_AC | 8%Pt | AA | λ>420 nm | 3190.00 | [11] |
| DNCA-2_AC |  |  |  | 3313.33 |  |
| DNCA-3_AC |  |  |  | 1260.00 |  |
| COF-932 | 3%Pt | AA | 350–780 nm | 110 | [12] |
| COF-932-AC |  |  |  | 730 |  |
| PyTz -COF | 3%Pt | AA | AM 1.5 | 2072.4 | [13] |
| ZnP-Tt | Pt | BIH | λ>420 nm | 11072 | [14] |
| ZnP-Tp |  |  |  | 5116 |  |
| ZnP-Py |  |  |  | 2478 |  |
| ZnP-Pt |  |  |  | 1098 |  |
| TAPA-BTT-COF | 8 wt% Pt | AA | λ>420 nm | 3750 | [15] |
| TAPT-BTT-COF |  |  |  | 12160 |  |
| NKCOF-109 | Without Pt | AA | λ>420 nm | 500 | [16] |
| NKCOF-109 |  |  |  | 420 |  |
| NKCOF-110 |  |  |  | 400 |  |
| NKCOF-111 |  |  |  | 350 |  |
| NKCOF-108 | 5%Pt |  |  | 11600 |  |

**Table S3** Comparison of the oxidation potentials of different base sacrificial agent for photocatalytic reactions.

| **Base sacrificial agent** | **E^0^_OX_ (V vs. NHE)** | **Reference** |
| --- | --- | --- |
| BIH | 0.56 | [17] |
| TEOA | 0.84 | [18] |
| TEA | 0.93 | [19] |
| Na_2_S/Na_2_SO_3_ | -0.32 | [18] |
| Na_2_S | -0.77 | [20] |
| Na_2_SO_3_ | -0.93 | [21] |

**Table S4** Crystallographic Data of the PyAm-PhAl-COF.

| **PyAm-PhAl-COF** | | | |
| --- | --- | --- | --- |
| space group: P222 | | | |
| a=34.1273 Å, b=37.0549 Å, c=4.1767 Å  α=β=γ=90° | | | |
| Atom | x | y | z |
| C1 | 0.91438 | 0.96569 | 0.01116 |
| C2 | 0.95712 | 0.96564 | 0.00035 |
| C3 | 0.97924 | 0.9323 | -0.01137 |
| C4 | 0.41438 | 0.46569 | -0.01116 |
| C5 | 0.45712 | 0.46564 | -0.00035 |
| C6 | 0.47924 | 0.4323 | 0.01137 |
| C7 | 0.1108 | 0.931 | -0.03595 |
| C8 | 0.3892 | 0.569 | 0.03595 |
| C9 | 0.1468 | 0.92797 | 0.14251 |
| C10 | 0.17063 | 0.89594 | 0.12255 |
| C11 | 0.15849 | 0.86556 | -0.07128 |
| C12 | 0.12329 | 0.86879 | -0.25486 |
| C13 | 0.10052 | 0.90156 | -0.24682 |
| C14 | 0.3532 | 0.57203 | -0.14252 |
| C15 | 0.32937 | 0.60406 | -0.12255 |
| C16 | 0.34151 | 0.63444 | 0.07128 |
| C17 | 0.37671 | 0.63121 | 0.25486 |
| C18 | 0.39948 | 0.59844 | 0.24682 |
| N19 | 0.18021 | 0.83087 | -0.08585 |
| C20 | 0.21403 | 0.82304 | 0.05684 |
| C21 | 0.23222 | 0.78563 | 0.02475 |
| N22 | 0.31979 | 0.66913 | 0.08584 |
| C23 | 0.26941 | 0.77839 | 0.17248 |
| C24 | 0.28708 | 0.74311 | 0.14812 |
| C25 | 0.26778 | 0.71437 | -0.02476 |
| C26 | 0.23059 | 0.72161 | -0.17249 |
| C27 | 0.21292 | 0.75689 | -0.14812 |
| C28 | 0.28597 | 0.67696 | -0.05684 |
| H29 | 0.96512 | 0.90541 | -0.03908 |
| H30 | 0.46512 | 0.40541 | 0.03908 |
| H31 | 0.156 | 0.94989 | 0.30806 |
| H32 | 0.19743 | 0.89495 | 0.26988 |
| H33 | 0.11397 | 0.84608 | -0.41172 |
| H34 | 0.07491 | 0.90383 | -0.40779 |
| H35 | 0.344 | 0.55011 | -0.30806 |
| H36 | 0.30257 | 0.60505 | -0.26988 |
| H37 | 0.38603 | 0.65392 | 0.41172 |
| H38 | 0.42508 | 0.59617 | 0.40779 |
| H39 | 0.2296 | 0.84354 | 0.20262 |
| H40 | 0.28474 | 0.80004 | 0.30853 |
| H41 | 0.31576 | 0.73834 | 0.26538 |
| H42 | 0.21526 | 0.69996 | -0.30854 |
| H43 | 0.18424 | 0.76166 | -0.26539 |
| H44 | 0.2704 | 0.65646 | -0.20262 |
| H45 | 0.36172 | 0.5 | 0 |
| C46 | 0.3942 | 0.5 | 0 |
| C47 | 0.4785 | 0.5 | 0 |
| C48 | 0.8942 | 0 | 0 |
| C49 | 0.9785 | 0 | 0 |
| H50 | 1.13828 | 1 | 1 |

**Table S5** Crystallographic Data of the PyAl-PhAm-COF.

| **PyAl-PhAm-COF** | | | |
| --- | --- | --- | --- |
| space group: P222 | | | |
| a= 33.4760 Å, b= 36.5247 Å, c=4.1300 Å  α=β=γ=90° | | | |
| Atom | x | y | z |
| C1 | 0.91353 | 0.96584 | 0.03025 |
| C2 | 0.95668 | 0.96579 | 0.00907 |
| C3 | 0.97904 | 0.93253 | -0.00483 |
| C4 | 0.41353 | 0.46584 | -0.03025 |
| C5 | 0.45668 | 0.46579 | -0.00907 |
| C6 | 0.47904 | 0.43253 | 0.00483 |
| C7 | 0.11124 | 0.93114 | -0.08871 |
| C8 | 0.38876 | 0.56886 | 0.08871 |
| C9 | 0.14889 | 0.9258 | 0.07115 |
| C10 | 0.17077 | 0.89264 | 0.02858 |
| C11 | 0.1552 | 0.86379 | -0.17047 |
| C12 | 0.11879 | 0.86963 | -0.34136 |
| C13 | 0.09796 | 0.90329 | -0.31006 |
| C14 | 0.35111 | 0.5742 | -0.07115 |
| C15 | 0.32923 | 0.60736 | -0.02858 |
| C16 | 0.3448 | 0.63621 | 0.17047 |
| C17 | 0.38121 | 0.63037 | 0.34136 |
| C18 | 0.40204 | 0.59671 | 0.31006 |
| C19 | 0.17571 | 0.82731 | -0.20125 |
| N20 | 0.20727 | 0.81915 | -0.02153 |
| C21 | 0.2284 | 0.78401 | -0.01754 |
| C22 | 0.32429 | 0.67269 | 0.20125 |
| C23 | 0.26643 | 0.78245 | 0.13625 |
| C24 | 0.28807 | 0.74897 | 0.1523 |
| C25 | 0.2716 | 0.71599 | 0.01754 |
| C26 | 0.23357 | 0.71755 | -0.13625 |
| C27 | 0.21193 | 0.75103 | -0.15229 |
| N28 | 0.29273 | 0.68085 | 0.02153 |
| H29 | 0.96478 | 0.90563 | -0.027 |
| H30 | 0.46478 | 0.40563 | 0.027 |
| H31 | 0.16066 | 0.94641 | 0.24256 |
| H32 | 0.19904 | 0.88909 | 0.16178 |
| H33 | 0.10667 | 0.84829 | -0.5043 |
| H34 | 0.071 | 0.90729 | -0.45794 |
| H35 | 0.33934 | 0.55359 | -0.24256 |
| H36 | 0.30096 | 0.61091 | -0.16178 |
| H37 | 0.39333 | 0.65171 | 0.5043 |
| H38 | 0.429 | 0.59271 | 0.45794 |
| H39 | 0.27945 | 0.80744 | 0.24321 |
| H40 | 0.31767 | 0.74944 | 0.26681 |
| H41 | 0.22055 | 0.69256 | -0.24321 |
| H42 | 0.18233 | 0.75056 | -0.26681 |
| H43 | 0.16332 | 0.80733 | -0.3752 |
| H44 | 0.33668 | 0.69267 | 0.37521 |
| H45 | 0.36032 | 0.5 | 0 |
| C46 | 0.39305 | 0.5 | 0 |
| C47 | 0.47828 | 0.5 | 0 |
| C48 | 0.89305 | 0 | 0 |
| C49 | 0.97828 | 0 | 0 |
| H50 | 1.13968 | 1 | 1 |

**References**

1. M. Frisch, G. Trucks, H. Schlegel, G. Scuseria, M. Robb, Gaussian 16, Revision B.01, Gaussian, Inc.: Wallingford, CT, **2016**.
2. A. Marenich, C. Cramer, D. Truhlar, “Universal Solvation Model Based on Solute Electron Density and on a Continuum Model of the Solvent Defined by the Bulk Dielectric Constant and Atomic Surface Tensions”, *J. Phys. Chem. B.* **2009**, *113*, 6378-6396.
3. A. Becke, “Density-functional thermochemistry. III. The role of exact exchange”, *J. Chem. Phys.* **1993**, *98*, 5648-5652.
4. C. Lee, W. Yang, R. Parr, “Development of the colle-salvetti correlation-energy formula into a functional of the electron-density”, *Phys. Rev. B.* **1988**, *37*, 785-789.
5. P. Stephens, F. Devlin, C. Chabalowski, M. Frisch, “Ab-initio calculation of vibrational absorption and circular-dichroism spectra using density-functional force-fields”, *J. Phys. Chem.* **1994**, *98*, 11623-11627.
6. S. Grimme, J. Antony, S. Ehrlich, H. Krieg, “A consistent and accurate ab initio parametrization of density functional dispersion correction (DFT-D) for the 94 elements H-Pu”, *J. Chem. Phys.* **2010**, *132*, 154104.
7. W. Dong, X. Yu, Z. Qin, Y. Chen, S. Ren, L. Li, “Synergistic Enhancement of Photocatalytic Hydrogen Evolution in Covalent Organic Frameworks via Isoreticular Design, Isomerism, and Protonation”, *Angew. Chem. Int. Ed.* **2025**, *64*, e202511200.
8. Y. Zhong, W. Dong, S. Ren, L. Li, “Oligo(Phenylenevinylene)-Based Covalent Organic Frameworks with Kagome Lattice for Boosting Photocatalytic Hydrogen Evolution”, *Adv. Mater.* **2024**, *36*, 2308251.
9. X. Ren, J. Sun, Y. Li, F. Bai, “Primitive functional groups directed distinct photocatalytic performance of imine-linked donor-acceptor covalent organic frameworks”, *Nano Res.* **2024**, *17*, 4994-5001.
10. H. He, R. Shen, P. Zhang, G. Liang, X. Li, “Inducing local charge polarization by constructing isomeric covalent organic frameworks with different orientations of imine bonds for enhancing photocatalytic hydrogen evolution”, *J. Mater. Chem. A.* **2024**, *12*, 227-232.
11. J. Yang, S. Ghosh, J. Roeser, A. Acharjya, C. Penschke, Y. Tsutsui, J. Rabeah, T. Wang, S. Y. D. Tameu, M.-Y. Ye, J. Grüneberg, S. Li, C. Li, R. Schomäcker, R. Van De Krol, S. Seki, P. Saalfrank, A. Thomas, “Constitutional isomerism of the linkages in donor–acceptor covalent organic frameworks and its impact on photocatalysis”, *Nat. Commun.* **2022**, *13*, 6317.
12. W. Dong, Z. Qin, K. Wang, Y. Xiao, X. Liu, S. Ren, L. Li, “Isomeric Oligo(Phenylenevinylene)-Based Covalent Organic Frameworks with Different Orientation of Imine Bonds and Distinct Photocatalytic Activities”, *Angew. Chem. Int. Ed.* **2023**, *62*, e202216073.
13. W. Li, X. Huang, T. Zeng, Y. A. Liu, W. Hu, H. Yang, Y.-B. Zhang, K. Wen, “Thiazolo[5,4-d]thiazole-Based Donor–Acceptor Covalent Organic Framework for Sunlight-Driven Hydrogen Evolution”, *Angew. Chem. Int. Ed.* **2021**, *60*, 1869-1874.
14. L. Zou, D. Si, S. Yang, Z. Chen, Y. Huang, R. Cao, “Induced Charge-Compensation Effect for Boosting Photocatalytic Water Splitting in Covalent Organic Frameworks”, *Angew. Chem. Int. Ed.* **2025**, *137*, e202418319.
15. X. Liu, X. Yang, X. Ding, H. Wang, W. Cao, Y. Jin, B. Yu, J. Jiang, “Covalent Organic Frameworks with Imine Proton Acceptors for Efficient Photocatalytic H2 Production”, *Chin. Chem. Lett.* **2023**, *34*, 108148.
16. Z. Zhao, Y. Zheng, C. Wang, S. Zhang, J. Song, Y. Li, S. Ma, P. Cheng, Z. Zhang, Y. Chen, “Fabrication of Robust Covalent Organic Frameworks for Enhanced Visible-Light-Driven H_2_ Evolution”, *ACS Catal.* **2021**, *11*, 2098-2107.
17. Z.-L. Xie, N. Gupta, J. Niklas, O. G. Poluektov, V. M. Lynch, K. D. Glusac, K. L. Mulfort, “Photochemical charge accumulation in a heteroleptic copper(I)-anthraquinone molecular dyad via proton-coupled electron transfer”, *Chem. Sci.* **2023**, *14*, 10219-10235.
18. C. Zhang, Z.-C. Shao, X.-L. Zhang, G.-Q. Liu, Y.-Z. Zhang, L. Wu, C.-Y. Liu, Y. Pan, F.-H. Su, M.-R. Gao, Y. Li, S.-H. Yu, “Design principles for maximizing hole utilization of semiconductor quantum wires toward efficient photocatalysis”, *Angew. Chem. Int. Ed.* **2023**, *62*, e202305571.
19. S. Biswas, F. A. Rahimi, R. K. Saravanan, A. Dey, J. Chauhan, D. Surendran, S. Nath, T. K. Maji, “A triazole-based covalent organic framework as a photocatalyst toward visible-light-driven CO_2_ reduction to CH_4_”, *Chem. Sci.* **2024**, *15*, 16259-16270.
20. Bessekhouad, Y., and Trari, M. “Photocatalytic hydrogen production from suspension of spinel powders AMn2O4 (A= Cu and Zn).” *Int. J. Hydrogen Energy* **2002**, *27*(4), 357-362.
21. Gu, J.-X., Chen, H., Ren, Y., Gu, Z.-G., Li, G., Xu, W.-J., Yang, X.-Y., Wen, J.-X., Wu, J.-T., and Jin, H.-G. “A Novel Cerium(IV)-Based Metal-Organic Framework for CO_2_ Chemical Fixation and Photocatalytic Overall Water Splitting.” *ChemSusChem* **2022**, *15*(1), e202102368.
